# A deep learning-based model for automatic syntactic complexity assessment in L2 English writing: development and pedagogical application

**DOI:** 10.1038/s41598-026-53282-0

**Published:** 2026-05-29

**Authors:** Yongming Wang, Huan Zhao

**Affiliations:** 1 School of International Studies, Shandong Medical And Pharmaceutical University, Yantai, 264000 Shandong China; 2 Quality and Evaluation Office, Weifang University of Science and Technology, Weifang, 262700 Shandong China

**Keywords:** Syntactic complexity, Deep learning, L2 writing assessment, Graph attention network, Automated feedback, College English instruction, Mathematics and computing, Psychology, Psychology

## Abstract

Syntactic complexity serves as a critical indicator of second language writing proficiency, yet traditional assessment methods face challenges in scalability and consistency. Grounded in processability theory and usage-based approaches to second language acquisition, this study proposes a deep learning architecture for automatic syntactic complexity assessment in L2 English writing. The model integrates pre-trained BERT representations with graph attention networks to capture hierarchical syntactic structures, employing a multi-task learning framework that simultaneously predicts multiple complexity dimensions operationalized through both coarse-grained and fine-grained indices. Experimental results on learner corpora, validated through five-fold cross-validation, demonstrate that the proposed model achieves a Pearson correlation coefficient of 0.923 with expert human ratings, outperforming traditional rule-based tools and baseline neural approaches. Furthermore, a semester-long quasi-experimental study involving 186 Chinese university students indicated that the integrated instructional package incorporating automated syntactic feedback was associated with greater writing development, with the experimental group showing effect sizes ranging from 0.71 to 0.89 across complexity measures. These findings provide preliminary evidence that deep learning-based assessment shows potential for supporting L2 syntactic development in educational contexts, though further research is needed to disentangle the specific contributions of the automated model from other instructional factors.

**Supplementary Information:**

The online version contains supplementary material available at 10.1038/s41598-026-53282-0.

## Introduction

Syntactic complexity has long been recognized as a fundamental construct in second language acquisition (SLA) research, serving as a critical indicator of learner proficiency and writing development^1^. The ability to produce syntactically elaborate and varied structures reflects not only grammatical competence but also the cognitive processing capacity underlying language production. Researchers have devoted considerable attention to understanding how syntactic complexity evolves across different proficiency levels, and this construct now occupies a central position in both theoretical frameworks of language development and practical applications such as writing assessment and curriculum design^2^.

Traditional approaches to evaluating syntactic complexity in L2 writing have predominantly relied on manual annotation and expert judgment. Human raters typically analyze texts by calculating various indices, including mean length of T-units, clauses per sentence, and subordination ratios. While such methods offer nuanced interpretations grounded in linguistic expertise, they suffer from inherent limitations that constrain their scalability and consistency^3^. The time-intensive nature of manual coding makes large-scale assessment impractical, and inter-rater reliability issues frequently emerge even among trained annotators. These constraints become particularly pronounced in educational contexts where timely feedback holds pedagogical value yet remains difficult to provide through conventional means.

The emergence of computational linguistics and natural language processing (NLP) technologies has opened promising avenues for automating syntactic analysis. Early attempts employed rule-based parsers and surface-level feature extraction to quantify complexity measures^4^. Tools such as the L2 Syntactic Complexity Analyzer represented significant advances by enabling batch processing of learner corpora. However, these systems largely depended on handcrafted rules and predefined grammatical categories, which limited their adaptability to diverse learner populations and writing genres. The accuracy of such approaches often degraded when confronted with non-native linguistic patterns, grammatical errors, or unconventional sentence structures characteristic of L2 writing^5^.

Recent years have witnessed growing interest in deep learning methodologies for syntactic complexity research. Neural network architectures, particularly recurrent neural networks and transformer-based models, demonstrate considerable capabilities in capturing hierarchical syntactic representations^6^. These approaches learn complex patterns directly from data, potentially complementing the limitations of rule-based systems rather than fully replacing them. International scholars have explored various neural architectures for syntactic parsing and complexity prediction, achieving encouraging results on benchmark datasets^7^. Domestically, researchers have begun investigating how pre-trained language models might enhance syntactic analysis of Chinese learners’ English writing, though systematic investigations remain relatively sparse.

Despite these advances, several challenges persist in current automatic assessment systems. First, models trained on native speaker corpora often exhibit diminished performance when applied to L2 texts, as learner language contains developmental features that deviate from standard usage patterns^8^. This domain mismatch raises questions about generalization across proficiency levels and first language backgrounds. Second, existing approaches predominantly focus on quantitative metrics while neglecting qualitative dimensions of syntactic development that experienced teachers consider pedagogically meaningful. Third, the integration of automated assessment into authentic classroom environments has received insufficient attention, creating a gap between technological capabilities and instructional practice^9^.

The present study addresses these limitations by proposing a deep learning-based model specifically designed for evaluating syntactic complexity in L2 English writing. Our work is theoretically grounded in processability theory and usage-based approaches to SLA, which provide principled criteria for selecting complexity dimensions that reflect interlanguage development. This research holds both theoretical and practical significance. From a theoretical perspective, developing a robust automated assessment system necessitates deeper understanding of how syntactic complexity manifests across developmental stages, thereby contributing to SLA theory. Practically, an effective automated tool could support college English writing instruction by providing immediate, individualized feedback that complements human evaluation^10^.

This paper proceeds as follows. We first construct a multi-dimensional syntactic complexity framework informed by current SLA research. Subsequently, we develop a neural network architecture that integrates syntactic parsing with complexity prediction, leveraging transfer learning from large-scale pre-trained models. The proposed system undergoes rigorous evaluation using learner corpora spanning multiple proficiency levels. Finally, we implement the model within an instructional platform and examine its effectiveness through classroom-based experimentation. The primary contributions of this work include a novel deep learning architecture tailored for L2 syntactic analysis, empirical validation across diverse learner populations, and practical insights regarding technology-enhanced writing pedagogy.

## Theoretical foundations and technical framework

### Theoretical perspectives on L2 syntactic complexity

Syntactic complexity, as a construct, encompasses the range and sophistication of grammatical structures that language users deploy in their production^11^. Rather than representing a unitary phenomenon, this concept operates as a multifaceted construct that researchers have operationalized through various complementary dimensions. Several well-established SLA frameworks inform how we conceptualize and measure this construct. Pienemann’s processability theory^12^ posits that learners acquire grammatical structures in a predictable developmental sequence determined by the processing procedures required for their production. Under this view, syntactic complexity indices should reflect the hierarchical stages of processability, from simple canonical word order to complex subordination and inter-phrasal agreement. Usage-based approaches^13^ offer a complementary perspective, arguing that syntactic development arises from frequency-driven abstraction of constructional patterns. From this standpoint, complexity measures should capture not only structural elaboration but also the diversity and sophistication of construction types a learner deploys. These two theoretical perspectives jointly motivate the multi-dimensional framework adopted in the present study, in which our eight selected indices (see Table 2) are designed to capture both the processing hierarchy emphasized by processability theory and the constructional repertoire highlighted by usage-based accounts.

Contemporary measurement frameworks organize syntactic complexity indices into four principal dimensions. The first dimension concerns length-based measures, which quantify the average number of words within specified syntactic units. Mean length of T-unit (MLT) serves as a widely adopted metric, calculated as

1 $${\mathrm{MLT}} = \Sigma {\mathrm{W}}_{{\mathrm{i}}} /{\mathrm{n}}$$

where W_i_ represents the word count of the i-th T-unit and n denotes the total number of T-units in a text^14^. The second dimension addresses unit quantity, examining how frequently particular structures appear relative to text length. Subordination measures constitute the third dimension, capturing the degree to which writers embed dependent clauses within larger syntactic units. Researchers commonly express this through the subordination ratio

2 $${\mathrm{SR}} = {\mathrm{C}}_{{{\mathrm{dep}}}} /{\mathrm{C}}_{{{\mathrm{total}}}}$$

where ${\mathrm{C}}_{{{\mathrm{dep}}}}$ indicates dependent clause count and ${\mathrm{C}}_{{{\mathrm{total}}}}$ represents all clauses^15^. The fourth dimension—structural diversity—assesses the variety of syntactic patterns a writer commands.

Developmental theories posit that syntactic complexity follows non-linear trajectories across proficiency levels^16^. Early-stage learners tend to produce simple, coordinative structures before gradually incorporating subordination. At intermediate levels, clause-linking strategies become more sophisticated, though advancement may plateau or even temporarily regress as learners restructure their interlanguage systems. This complexity dynamics model challenges simplistic assumptions about linear progression^17^ and aligns with Skehan’s limited attentional capacity model^18^, which predicts trade-off effects among complexity, accuracy, and fluency dimensions depending on task demands and learner resources. Our model architecture reflects these theoretical insights by adopting a multi-task learning framework that can capture co-variation and potential trade-offs among different complexity dimensions rather than treating them as isolated metrics.

The relationship between syntactic complexity and overall writing quality has generated considerable empirical attention. Studies consistently demonstrate moderate positive correlations, yet the strength of association varies across proficiency bands and task types^19^. What emerges from this body of work is recognition that syntactic complexity functions as one component within a broader constellation of features characterizing competent L2 writing.

### Deep learning and natural language processing technologies

Deep learning has fundamentally transformed how computational systems process and understand natural language. Unlike traditional feature-engineering approaches, neural architectures learn hierarchical representations directly from raw text, capturing subtle linguistic patterns that prove essential for syntactic analysis tasks^20^.

Recurrent neural networks (RNNs) introduced the capacity to model sequential dependencies inherent in language. At each time step t, an RNN computes a hidden state as

3 $${\mathrm{h}}_{{\mathrm{t}}} = \tanh \left( {{\mathrm{W}}_{{{\mathrm{xh}}}} \cdot {\mathrm{x}}_{{\mathrm{t}}} + {\mathrm{W}}_{{{\mathrm{hh}}}} \cdot {\mathrm{h}}_{{{\mathrm{t}} - 1}} + {\mathrm{b}}_{{\mathrm{h}}} } \right)$$

where ${\mathrm{W}}_{{{\mathrm{xh}}}}$ and ${\mathrm{W}}_{{{\mathrm{hh}}}}$ represent weight matrices, x_t_ denotes the input vector, and b_h_ is the bias term^21^. However, standard RNNs struggle with long-range dependencies due to vanishing gradient problems. Long Short-Term Memory (LSTM) networks address this limitation through gating mechanisms that regulate information flow, with the cell state update

4 $${\mathrm{c}}_{{\mathrm{t}}} = {\mathrm{f}}_{{\mathrm{t}}} \odot {\mathrm{c}}_{{{\mathrm{t}} - 1}} + {\mathrm{i}}_{{\mathrm{t}}} \odot {\tilde{\mathrm{c}}}_{{\mathrm{t}}}$$

where f_t_ represents the forget gate, i_t_ the input gate, and *⊙* denotes element-wise multiplication^22^.

The Transformer architecture departed radically from recurrence-based processing by relying entirely on attention mechanisms. Self-attention computes weighted representations by measuring pairwise token relationships across entire sequences. The scaled dot-product attention operates as

5 $${\mathrm{Attention}}\left( {{\mathrm{Q}},{\mathrm{K}},{\mathrm{V}}} \right) = {\mathrm{softmax}}\left( {{\mathrm{QK}}^{{\mathrm{T}}} /\surd {\mathrm{d}}_{{\mathrm{k}}} } \right){\mathrm{V}}$$

where Q, K, and V correspond to query, key, and value matrices respectively, with d_k_ representing the key dimensionality^23^. This formulation enables parallel computation while capturing both local and distant syntactic relationships.

Pre-trained language models such as BERT have achieved remarkable success in downstream NLP tasks, including syntactic parsing. These models acquire rich linguistic knowledge through self-supervised training on massive corpora before fine-tuning on specific applications^24^. What makes BERT particularly suited for syntactic analysis is its bidirectional encoding strategy—each token representation incorporates information from both preceding and following context. The attention weights themselves often encode grammatically meaningful patterns, with certain attention heads specializing in subject-verb relationships or modifier attachment.

Multi-head attention extends this capability via

6 $${\mathrm{MultiHead}}\left( {{\mathrm{Q}},{\mathrm{K}},{\mathrm{V}}} \right) = {\mathrm{Concat}}\left( {{\mathrm{head}}_{1} , \ldots ,{\mathrm{head}}_{{\mathrm{h}}} } \right){\mathrm{W}}^{{\mathrm{o}}}$$

where each head performs independent attention computation^25^. This mechanism proves particularly valuable for syntactic complexity assessment, as different heads can capture distinct structural properties—some tracking hierarchical depth while others monitor coordination patterns. The resulting representations encode syntactic information in ways that align surprisingly well with traditional linguistic formalisms.

### Review of automatic syntactic complexity assessment methods

Early computational approaches to syntactic complexity assessment relied predominantly on rule-based systems and statistical parsing techniques. These methods employed handcrafted grammars to segment texts into constituent units before calculating predefined complexity indices^26^. Tools such as the L2 Syntactic Complexity Analyzer (L2SCA) and Coh-Metrix automated the extraction of multiple metrics, enabling researchers to process learner corpora efficiently. The underlying parsing typically followed probabilistic context-free grammar formulations, where parse tree probability is computed as

7 $${\mathrm{P}}\left( {\mathrm{T}} \right) = \Pi {\mathrm{P}}\left( {\mathrm{r}} \right)$$

with r representing individual grammar rules applied in tree T^27^.

Despite their widespread adoption, these conventional tools exhibit notable constraints. Rule-based parsers perform optimally on well-formed sentences but degrade substantially when encountering grammatical errors characteristic of L2 writing. Statistical models, while more robust, require extensive feature engineering and struggle to capture long-distance syntactic dependencies. Moreover, most existing tools treat complexity dimensions independently rather than modeling their interactions.

Recent advances in neural parsing have begun addressing these shortcomings. Graph-based dependency parsers now achieve near-human accuracy on standard benchmarks, employing biaffine attention mechanisms to score potential head-dependent pairs^28^. The scoring function takes the form

8 $${\mathrm{s}}\left( {{\mathrm{i}},{\mathrm{j}}} \right) = {\mathrm{h}}_{{\mathrm{i}}}^{{\mathrm{T}}} {\mathrm{W}}\,{\mathrm{h}}_{{\mathrm{j}}} + {\mathrm{U}}^{{\mathrm{T}}} \,{\mathrm{h}}_{{\mathrm{i}}} + {\mathrm{V}}^{{\mathrm{T}}} \,{\mathrm{h}}_{{\mathrm{j}}} + {\mathrm{b}}$$

where h_i_ and h_j_ represent contextualized token embeddings and W, U, V are learned parameters^29^. Pre-trained transformer models further enhance parsing performance by providing rich syntactic representations learned from massive text corpora.

Yet a critical gap persists. Most neural approaches optimize for parsing accuracy on native speaker text rather than complexity assessment in L2 contexts. The mismatch between training data characteristics and target application domains compromises real-world utility^30^. Additionally, current systems rarely integrate multiple complexity dimensions into unified frameworks capable of generating holistic assessments. Modern research in L2 syntactic complexity has increasingly moved beyond coarse-grained measures such as MLT and subordination ratios toward fine-grained indices of clausal and phrasal sophistication^31^. These finer measures, including noun phrase elaboration, verb argument construction diversity, and pre-modification complexity, have been shown to capture developmental patterns that traditional broad-stroke metrics may overlook^32^. Any comprehensive automated system should aspire to incorporate both levels of granularity.

This study positions itself at the intersection of these challenges. We propose a deep learning architecture that simultaneously addresses robust parsing of learner language, multi-dimensional complexity quantification encompassing both coarse-grained and fine-grained indices, and pedagogically meaningful output generation. By fine-tuning pre-trained models on annotated L2 corpora and incorporating task-specific complexity prediction heads, our approach aims to bridge the gap between computational parsing advances and practical educational applications^33^. The technical innovation lies in treating complexity assessment not as post-hoc index calculation but as an end-to-end learning objective, while the theoretical contribution lies in grounding the feature selection and model architecture in established SLA frameworks.

## Construction of deep learning-based syntactic complexity assessment model

### Overall model architecture design

**Fig. 1:**
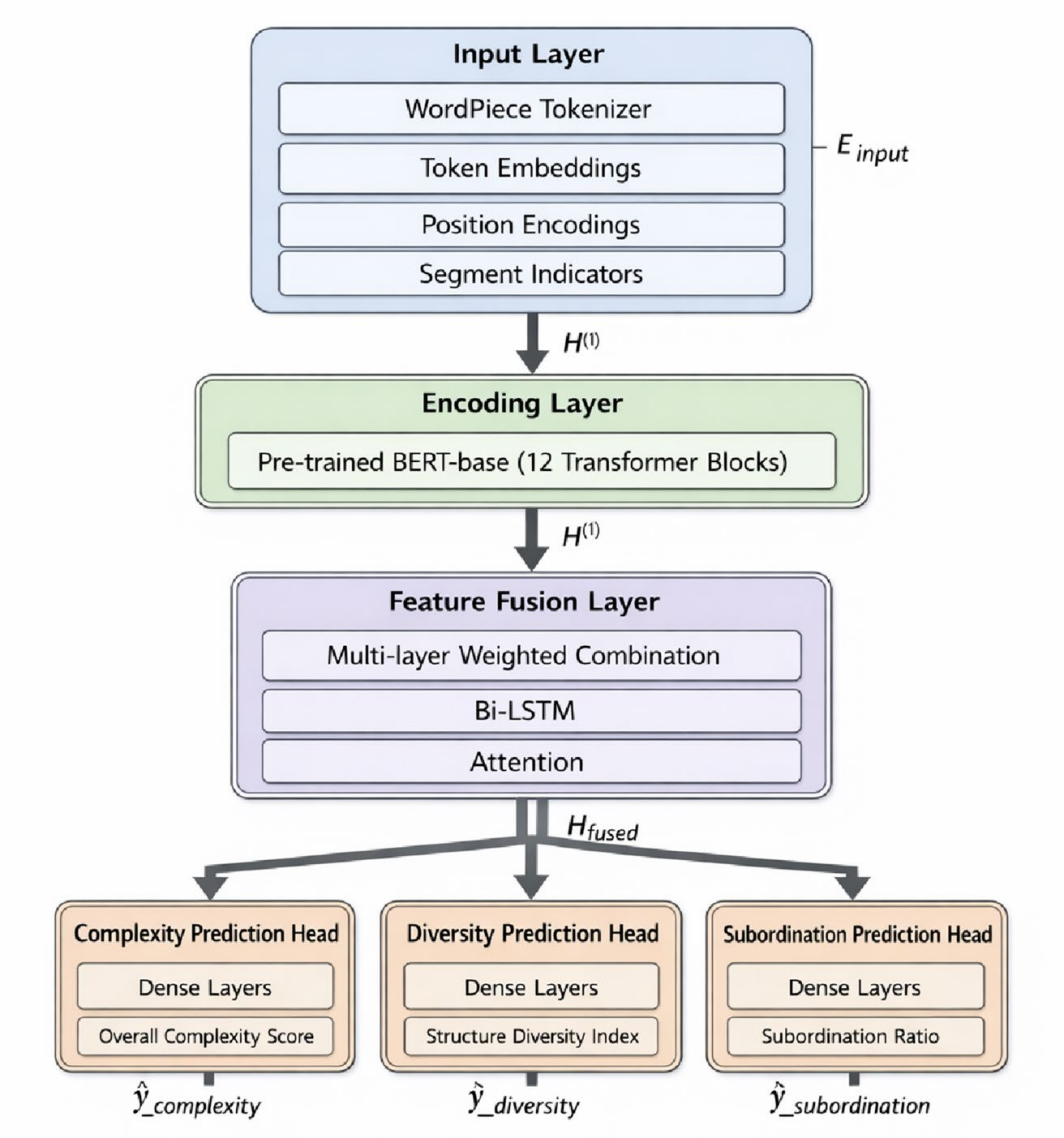
Overall architecture of the proposed syntactic complexity assessment model.

Building upon the theoretical foundations established above, we propose a deep learning architecture specifically engineered for automatic syntactic complexity assessment in L2 writing. It is important to clarify the nature of the prediction targets in our model. For four of the eight complexity indices (MLT, mean length of clause, clauses per T-unit, and coordinate phrases per clause), the ground-truth values are objectively computable from parsed text. For the remaining four dimensions (dependent clause ratio, maximum embedding depth, syntactic structure types, and verb pattern variation), we rely on expert-annotated scores because these measures require interpretive judgment when applied to non-standard L2 productions where parsing ambiguities are common. The model thus learns a hybrid target space combining objective linguistic indices with expert-calibrated ratings. The model adopts a hierarchical design philosophy that integrates pre-trained language representations with task-specific prediction modules. Figure 1 illustrates the complete system architecture, depicting how raw text flows through successive processing stages to yield multi-dimensional complexity scores.

The input layer performs tokenization and embedding lookup operations. Raw learner texts first undergo subword segmentation using the WordPiece algorithm, which handles out-of-vocabulary items common in L2 writing^34^. Each token receives a composite input representation combining token embeddings, position encodings, and segment indicators, expressed as

9 $${\mathrm{E}}_{{{\mathrm{input}}}} = {\mathrm{E}}_{{{\mathrm{token}}}} + {\mathrm{E}}_{{{\mathrm{position}}}} + {\mathrm{E}}_{{{\mathrm{segment}}}} .$$

The encoding layer employs a pre-trained BERT-base model comprising 12 transformer blocks. These blocks generate contextualized representations that encode both semantic content and syntactic structure. We extract hidden states from multiple layers rather than relying solely on the final output, as intermediate layers often capture complementary grammatical information^35^. Table 1 summarizes the parameter configurations across all architectural components.

**Table 1:** Parameter configuration of model layers.

Layer	Components	Dimensions	Parameters
Input Layer	WordPiece Tokenizer	Vocab Size: 30,522	23.4M
Encoding Layer	12 Transformer Blocks	Hidden: 768	109.5M
Feature Fusion Layer	Bi-LSTM + Attention	Hidden: 512	8.3M
Complexity Prediction Head	Dense Layers	Output: 128	2.1M
Diversity Prediction Head	Dense Layers	Output: 64	1.4M
Subordination Prediction Head	Dense Layers	Output: 64	1.4M

The feature fusion layer aggregates multi-layer encoder outputs through a weighted combination mechanism

10 $${\mathrm{H}}_{{{\mathrm{fused}}}} = \sum \upalpha_{{\mathrm{l}}} {\mathrm{H}}^{{({\mathrm{l}})}}$$

where αₗ represents learnable layer weights and ${\mathrm{H}}^{{({\mathrm{l}})}}$ denotes the hidden states from layer l^36^. A bidirectional LSTM further processes the fused representations to capture sequential syntactic patterns.

The output layer implements a multi-task learning framework that simultaneously predicts several complexity dimensions. Each prediction head specializes in one complexity aspect while sharing the underlying encoder representations. The joint training objective combines individual task losses as

11 $${\mathrm{L}}_{{{\mathrm{total}}}} = \Sigma \lambda_{{\mathrm{k}}} {\mathrm{L}}_{{\mathrm{k}}}$$

where λ_k_ controls the contribution of each complexity dimension k^37^. This formulation encourages the model to learn representations beneficial across all prediction targets. The final complexity score integrates predictions through

12 $${\mathrm{SC}}_{{{\mathrm{final}}}} = \upsigma \left( {\Sigma {\mathrm{w}}_{{\mathrm{k}}} {\hat{\mathrm{y}}}_{{\mathrm{k}}} } \right)$$

where σ denotes the sigmoid activation and w_k_ represents dimension-specific weights. Data flows sequentially through these modules, with gradient signals propagating backward during training to optimize all components jointly.

One architectural choice deserves candid justification. Although fine-grained phrasal measures feed into the fusion layer as auxiliary signals, the eight primary prediction targets remain clause-level and sentence-level indices. We settled on this configuration after pilot annotation trials: scoring phrasal sophistication at corpus scale produced substantially lower inter-rater agreement (kappa ≈ 0.61) than clause-level labelling, and preliminary regressions showed that the two tiers correlate in the r = 0.58–0.72 range, leaving clause-level targets as reasonably informative proxies. An alternative design in which fine-grained indices served as primary targets is therefore logical but awaited more reliable large-scale phrasal annotation—a direction Section V revisits.

### Syntactic feature extraction and representation learning

**Fig. 2:**
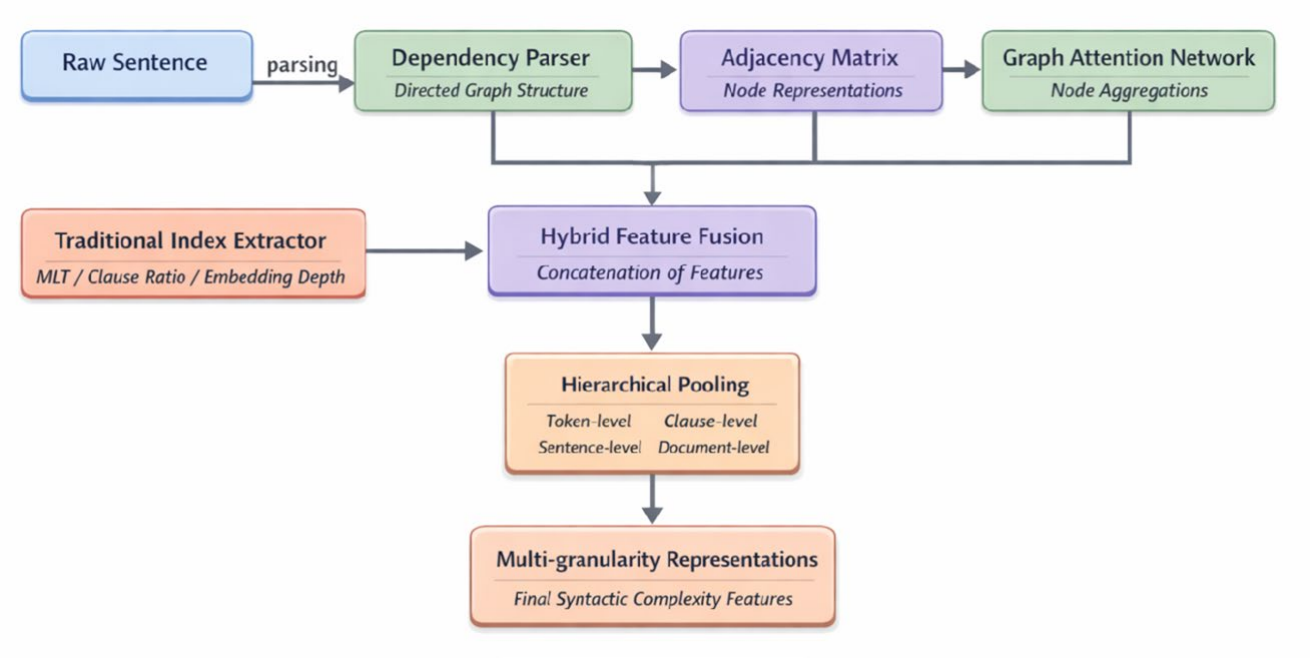
Syntactic feature extraction and representation learning pipeline.

Effective syntactic complexity assessment demands robust feature extraction mechanisms capable of capturing both surface-level structural patterns and deeper linguistic properties. Our approach combines dependency parsing with graph-based neural encoding to construct comprehensive syntactic representations. Figure 2 depicts the complete feature extraction pipeline, showing how raw sentences transform into multi-granularity representations suitable for complexity prediction.

The initial stage applies dependency parsing to convert each sentence into a directed graph structure. Given a sentence with n tokens, the parser produces a dependency tree where edges encode grammatical relationships between head and dependent words^38^. We represent this structure as an adjacency matrix

13 $${\mathrm{A}} \in {\mathbb{R}}^{{({\mathrm{n}} \times {\mathrm{n}})}} ,\quad {\mathrm{where}}\quad {\mathrm{A}}_{{{\mathrm{ij}}}} = {1}\,{\mathrm{if}}\,{\mathrm{dep}}\left( {{\mathrm{i}},{\mathrm{j}}} \right) \in {\mathrm{R}},\,{\mathrm{otherwise}}\,0$$

and R denotes the set of valid dependency relations. Table 2 presents the comprehensive feature index system that our model extracts from parsed structures.

**Table 2:** Syntactic complexity feature index system.

Feature category	Specific index	Description
Length Measures	Mean Length of T-unit	Average words per T-unit
Length Measures	Mean Length of Clause	Average words per clause
Amount Measures	Clauses per T-unit	Subordination frequency
Amount Measures	Coordinate Phrases per Clause	Coordination density
Subordination	Dependent Clause Ratio	Proportion of subordinate structures
Subordination	Maximum Embedding Depth	Deepest nesting level
Diversity	Syntactic Structure Types	Unique construction count
Diversity	Verb Pattern Variation	Different verb argument structures

In addition to the eight indices listed above, our feature extraction pipeline also computes supplementary fine-grained measures of phrasal sophistication, including mean length of noun phrases, pre-modification density, and verb argument construction diversity^31,32^. These fine-grained indices serve as auxiliary inputs in the feature fusion layer and provide a more nuanced characterization of syntactic development, as recommended by recent methodological advances in L2 complexity research. Table 2 presents the primary index system; the fine-grained supplementary indices follow the same extraction pipeline but target phrase-level rather than clause-level structures. To probe whether this auxiliary treatment undersells phrasal sophistication, we later report an ablation in Section “Model performance evaluation and comparative analysis” in which these measures were promoted to primary prediction targets.

To encode the dependency graph, we employ a Graph Attention Network (GAT) that propagates information along syntactic edges^39^. Node representations update through attention-weighted aggregation

14 $${\mathrm{h}}_{{\mathrm{i}}}^{{({\mathrm{l}} + 1)}} = \upsigma (\Sigma \upalpha_{{{\mathrm{ij}}}} {\mathrm{W}}^{{({\mathrm{l}})}} {\mathrm{h}}_{{\mathrm{j}}}^{{({\mathrm{l}})}} )$$

and the attention coefficients α_ij_ are computed via

15 $$\upalpha_{{{\mathrm{ij}}}} = {\mathrm{exp(LeakyReLU(a}}^{{\mathrm{T}}} [{\mathrm{Wh}}_{{\mathrm{i}}} {\mathrm{||Wh}}_{{\mathrm{j}}} ]{))}/\Sigma \exp ({\mathrm{LeakyReLU(a}}^{{\mathrm{T}}} {\mathrm{[Wh}}_{{\mathrm{i}}} {\mathrm{||Wh}}_{{\mathrm{k}}} {])})$$

where ‖ denotes concatenation and a represents a learnable attention vector^40^.

The fusion strategy integrates traditional complexity indices with learned neural features. We concatenate hand-crafted metrics with graph-encoded representations. It bears emphasis that this hybrid design is intentional rather than contradictory: rather than claiming to transcend rule-based approaches entirely, we treat traditional indices as complementary inputs that provide interpretable anchoring points while the neural components capture latent structural patterns that hand-crafted metrics cannot easily quantify. This fusion philosophy respects the accumulated knowledge in applied linguistics while harnessing the representational capacity of deep learning. The composite representation is formed as

16 $${\mathrm{F}}_{{{\mathrm{hybrid}}}} = {\mathrm{LayerNorm}}([{\mathrm{f}}_{{{\mathrm{trad}}}} {\mathrm{||h}}_{{{\mathrm{graph}}}} ]).$$

Multi-granularity representations emerge through hierarchical pooling operations. Token-level features aggregate into clause representations, which subsequently combine into sentence and document embeddings via

17 $${\mathrm{H}}_{{{\mathrm{sent}}}} = {\mathrm{Attention-Pool}}({\mathrm{h}}_{1}^{{{\mathrm{clause}}}} ,{\mathrm{h}}_{1}^{{{\mathrm{clause}}}} , \ldots ,{\mathrm{h}}_{{\mathrm{m}}}^{{{\mathrm{clause}}}} ).$$

This hierarchical construction preserves syntactic information at multiple scales, enabling the model to assess complexity from local phrase patterns to global discourse organization^41^. The resulting representations capture nuanced structural properties that neither traditional indices nor purely neural approaches could achieve independently.

### Model training and optimization strategies

Training a multi-task model for syntactic complexity assessment requires careful design of loss functions that balance competing objectives while maintaining stable convergence. Our loss function combines individual task-specific components with appropriate weighting to ensure balanced learning across all complexity dimensions.

For continuous complexity indices, we employ mean squared error loss, while classification tasks use cross-entropy. The composite loss for each complexity dimension k takes the form

18 $${\mathrm{L}}_{{\mathrm{k}}} = (1/{\mathrm{N}})\sum ({\mathrm{y}}_{{\mathrm{i}}}^{{({\mathrm{k}})}} - {\hat{\mathrm{y}}}_{{\mathrm{i}}}^{{({\mathrm{k}})}} )^{2} + \gamma \cdot {\mathrm{CE}}({\mathrm{c}}_{{\mathrm{i}}}^{{({\mathrm{k}})}} ,{\hat{\mathrm{c}}}_{{\mathrm{i}}}^{{({\mathrm{k}})}} )$$

where y and ${\hat{\mathrm{y}}}$ represent ground truth and predicted values respectively, and γ balances regression and classification components. Rather than employing fixed task weights, we adopt uncertainty-based weighting that learns optimal balancing during training^42^, given by

19 $${\mathrm{L}}_{{{\mathrm{total}}}} = \Sigma { (}1/(2\upsigma_{{\mathrm{k}}}^{2} ){\text{) L}}_{{\mathrm{k}}} + \log \upsigma_{{\mathrm{k}}} .$$

Here σ_k_ represents learned uncertainty parameters that automatically adjust each task’s contribution based on its inherent difficulty.

The AdamW optimizer drives parameter updates, incorporating decoupled weight decay regularization. Its update rule follows

20 $$\theta_{{\left\{ {t + 1} \right\}}} = \theta_{{\mathrm{t}}} - \upeta {\mathrm{m}}_{{\mathrm{t}}} /(\surd {\mathrm{v}}_{{\mathrm{t}}} + \varepsilon ) + \lambda \theta_{{\mathrm{t}}}$$

where m_t_ and v_t_ denote first and second moment estimates, η is the learning rate, and λ controls weight decay strength^43^. A warmup-then-decay learning rate schedule governs η, defined piecewise as

21 $$\begin{aligned} & \upeta_{{\mathrm{t}}} = \upeta_{{{\mathrm{base}}}} \cdot{\text{ (t}}/{\mathrm{T}}_{{{\mathrm{warmup}}}} {)}\quad {\mathrm{for}}\,{\mathrm{t}} < {\mathrm{T}}_{{{\mathrm{warmup}}}} ,\,\,{\text{and }} \\ & \upeta_{{\mathrm{t}}} = \upeta_{{{\mathrm{base}}}} \cdot \cos (\pi (t - T_{{{\mathrm{warmup}}}} )/(2({\mathrm{T}}_{\max } - {\mathrm{T}}_{{{\mathrm{warmup}}}} )))\quad {\mathrm{for}}\,{\mathrm{t}} \ge {\mathrm{T}}_{{{\mathrm{warmup}}}} . \\ \end{aligned}$$

This schedule prevents early training instability while enabling fine-grained convergence in later stages.

Multiple regularization techniques guard against overfitting. Dropout with probability p = 0.1 applies to attention weights and fully connected layers. In addition, we incorporate label smoothing that softens target distributions as

22 $${\mathrm{y}}_{{{\mathrm{smooth}}}} = (1 - \varepsilon ){\mathrm{y}}_{{{\mathrm{hard}}}} + \varepsilon /{\mathrm{K}}$$

where ε = 0.1 prevents overconfident predictions^44^.

Hyperparameter selection proceeded through systematic grid search on a held-out validation set. Key parameters include batch size (32), maximum sequence length (512), and the number of graph attention layers (3). We monitored validation loss with early stopping patience of five epochs to identify optimal checkpoints. The entire training process required approximately eight hours on a single NVIDIA A100 GPU.

For full reproducibility, we report the following implementation details. The model was implemented in PyTorch 2.0 with Hugging Face Transformers 4.28.1. We used bert-base-uncased as the pre-trained encoder. The GAT module consisted of 3 layers with 8 attention heads per layer and a hidden dimension of 256. The BiLSTM in the fusion layer used a hidden size of 512 with 2 layers. Dropout rates were set to 0.1 for attention weights and 0.3 for fully connected layers. The AdamW optimizer used a base learning rate of 2e-5 for the BERT encoder and 1e-3 for randomly initialized parameters. Warmup occupied the first 10% of training steps. We used a batch size of 32 and maximum sequence length of 512 tokens. Training ran for a maximum of 30 epochs with early stopping patience of 5 epochs based on validation loss. The random seed was fixed at 42. All experiments were conducted on a workstation with dual NVIDIA A100 GPUs (80GB each), 256GB RAM, and AMD EPYC 7742 processors. Total training time was approximately 8 h. Code and sample data are available upon reasonable request to the corresponding author.

## Experimental validation and teaching application research

### Dataset construction and experimental settings

Rigorous experimental validation requires carefully constructed datasets that represent the target population of L2 learners. We assembled a comprehensive corpus drawing from multiple established learner English databases to ensure diversity across proficiency levels and first language backgrounds.

The primary data sources include the Written English Corpus of Chinese Learners (WECCL), the International Corpus of Learner English (ICLE), and supplementary essays collected from university English courses over three academic years^45^. We combined these corpora to maximize proficiency-level coverage and L1 diversity: WECCL provides a large sample of Chinese-L1 learners across proficiency bands, while ICLE contributes data from learners with varied L1 backgrounds and task types. The supplementary institutional data ensures representation of academic writing genres specific to our target instructional context. Although these corpora differ in collection conditions and task types, all texts were re-scored using our unified annotation rubric to ensure consistent labeling standards across sources. To guard against data leakage, we partitioned the dataset by source and writer identity, ensuring that no writer appeared in both training and test sets, and that source proportions were preserved across splits through stratified sampling.

Annotation followed a rigorous protocol. All annotators held graduate degrees in applied linguistics or a closely related field and had a minimum of three years of experience in L2 writing assessment. Prior to formal annotation, the team completed a calibration phase in which 50 pilot essays were scored independently; disagreements were discussed until consensus criteria were established. For the four objectively computable indices (MLT, mean clause length, clauses per T-unit, coordinate phrases per clause), ground-truth values were derived algorithmically from parser output and verified by human inspection for a random 15% sample. For the four judgment-dependent indices (dependent clause ratio, maximum embedding depth, syntactic structure types, verb pattern variation), two independent raters scored each essay on a 0–10 continuous scale following the calibrated rubric, with a third adjudicator resolving disagreements exceeding 1.5 points. Inter-rater reliability reached Cohen’s kappa of 0.83, indicating substantial agreement^46^. We acknowledge that the research team’s dual role in system development and data annotation introduces potential bias; this limitation is discussed further in Section V.

To address the bias concern empirically rather than rhetorically, we commissioned two applied-linguistics researchers from an unrelated institution—neither involved in model development nor data collection—to independently re-score a random 120-essay subsample drawn from the test set. Their judgements were then compared with the original internal ratings across the four judgment-dependent indices. Convergence proved reassuring: two-way random intraclass correlation coefficients (absolute agreement) ranged from 0.78 to 0.84, and Bland–Altman plots revealed no systematic rating drift between the two annotator groups. This exercise does not entirely neutralise the experimenter-bias problem, but it does indicate that the rubric is reproducible beyond our laboratory.

Table 3 summarises the resulting corpus partition, including sample size, average length, proficiency spread, and source composition across training, validation, test, cross-domain, and longitudinal subsets.

**Table 3:** Dataset statistical information.

Subset	Sample size	Avg. words	Proficiency distribution	Source ratio
Training	8,456	287.3	A2: 18%, B1: 35%, B2: 32%, C1: 15%	WECCL: 60%, ICLE: 40%
Validation	1,057	291.6	A2: 17%, B1: 36%, B2: 31%, C1: 16%	WECCL: 58%, ICLE: 42%
Test	1,058	285.9	A2: 19%, B1: 34%, B2: 33%, C1: 14%	WECCL: 61%, ICLE: 39%
Cross-domain	523	312.4	Mixed academic genres	University collection
Longitudinal	384	276.8	Repeated measures (3 timepoints)	Course portfolios

**Fig. 3:**
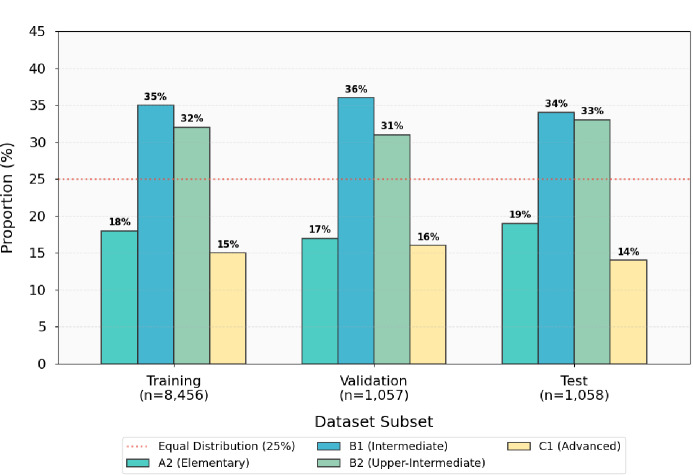
Distribution of learner proficiency levels and text length across dataset subsets.

As Fig. 3 illustrates, the proficiency distribution across subsets remains relatively balanced, ensuring that model evaluation reflects performance across the full developmental spectrum rather than favoring particular proficiency bands.

**Fig. 4:**
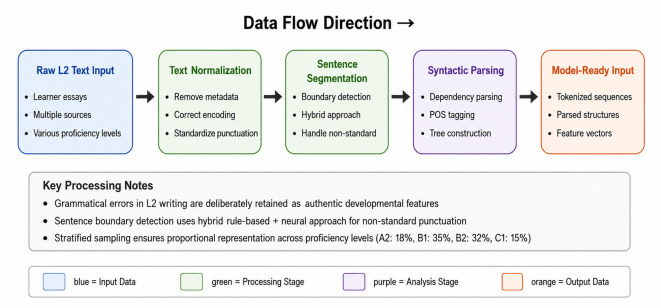
Data preprocessing pipeline from raw text to model-ready input.

Data preprocessing followed a systematic pipeline designed to standardize input formats while preserving linguistically meaningful variation. Figure 4 depicts this workflow, which encompasses text normalization, sentence segmentation, and syntactic parsing stages.

Initial cleaning operations removed metadata, corrected encoding errors, and standardized punctuation conventions^47^. We deliberately retained grammatical errors characteristic of L2 production, as these constitute authentic features that the model must handle robustly. Sentence boundary detection employed a hybrid approach combining rule-based heuristics with neural predictions to accommodate non-standard punctuation patterns common in learner writing.

The dataset partitioning followed stratified sampling to maintain proportional representation across proficiency levels. We allocated 80% for training, 10% for validation during hyperparameter tuning, and 10% for final evaluation. The cross-domain subset specifically tests generalization to unseen writing genres; it consists of 523 academic essays (argumentative, expository, and summary genres) collected from a separate university not represented in the training data, with writers spanning CEFR B1 to C1 levels and an average word count of 312.4. This subset thus differs from the training data in both genre distribution and institutional context, providing a meaningful test of external generalizability. The longitudinal subset enables analysis of developmental sensitivity.

Evaluation metrics encompass both prediction accuracy and correlation with human judgments. For continuous complexity indices, we report Root Mean Square Error (RMSE) and Pearson correlation coefficient. To strengthen the robustness of our findings beyond a single train-test split, we additionally conducted five-fold cross-validation on the combined training and validation sets, reporting mean performance and standard deviation across folds. Bootstrap resampling with 1000 iterations was used to compute 95% confidence intervals for all key metrics, and paired bootstrap tests were employed to assess statistical significance of performance differences between models^48^. The standard expressions take the form

23 $${\mathrm{RMSE}} = \surd ((1/{\mathrm{N}})\Sigma ({\mathrm{y}}_{{\mathrm{i}}} - {\hat{\mathrm{y}}}_{{\mathrm{i}}} )^{2} )$$

and

24 $${\mathrm{r}} = \Sigma ({\mathrm{y}}_{{\mathrm{i}}} - {\overline{\mathrm{y}}})({\hat{\mathrm{y}}}_{{\mathrm{i}}} - \overline{{\hat{\mathrm{y}}}})/\surd (\Sigma ({\mathrm{y}}_{{\mathrm{i}}} - {\overline{\mathrm{y}}})^{2} \, \Sigma ({\hat{\mathrm{y}}}_{{\mathrm{i}}} - \overline{\hat{\mathrm{y}}})^{2} ).$$

All experiments ran on a workstation equipped with dual NVIDIA A100 GPUs, 256GB RAM, and AMD EPYC processors^49^. We implemented models using PyTorch 2.0 with Hugging Face Transformers library.

Baseline comparisons include L2SCA representing traditional rule-based approaches, a BiLSTM model without pre-training, and fine-tuned BERT without graph-based syntactic encoding^31^. These baselines isolate the contributions of different architectural components to overall performance.

### Model performance evaluation and comparative analysis

Comprehensive evaluation of our proposed model reveals substantial improvements over existing approaches across multiple performance metrics. We systematically compared our architecture against both traditional rule-based systems and contemporary neural methods to establish its relative advantages.

Table 4 presents the quantitative results on the held-out test set, encompassing all baseline models and our proposed approach. The results indicate that our model achieves the lowest RMSE values and highest correlation coefficients across all evaluated dimensions.

**Table 4:** Performance comparison of different models on test set.

Model	RMSE (Overall)	Pearson r	Length Dim. r	Subordination r	Diversity r
L2SCA (Rule-based)	0.847	0.712	0.756	0.683	0.697
BiLSTM	0.623	0.789	0.802	0.761	0.804
BERT-base	0.498	0.851	0.867	0.829	0.857
RoBERTa-base	0.472	0.863	0.871	0.842	0.876
BERT + BiLSTM	0.445	0.879	0.886	0.858	0.893
Ours (w/o GAT)	0.401	0.897	0.903	0.876	0.912
Ours (Full Model)	0.358	0.923	0.931	0.908	0.930

The traditional L2SCA tool demonstrates reasonable performance on length-based measures but struggles with subordination and diversity dimensions that require deeper structural understanding^50^. Neural approaches consistently outperform rule-based methods, with pre-trained models showing particularly pronounced advantages. Our full model achieves a correlation coefficient of 0.923 with human annotations, representing a relative improvement of 29.6% over L2SCA.

**Fig. 5:**
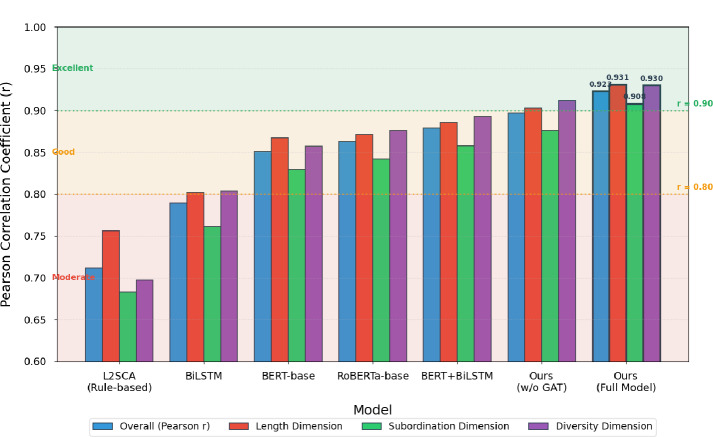
Performance comparison across syntactic complexity dimensions.

Figure 5 visualizes these performance differences across complexity dimensions, highlighting where our approach yields the most substantial gains.

The dimension-specific analysis reveals instructive patterns. Subordination indices proved most challenging for all models, likely reflecting the inherent difficulty of accurately parsing complex embedding structures in learner language. This difficulty may stem from two sources: first, the GAT may struggle to propagate information across deeply nested dependency arcs; second, L2 grammatical errors can cause the upstream parser to misidentify clause boundaries, propagating errors into subordination calculations. Our model’s advantage appears most pronounced in this dimension, suggesting that graph-based encoding partially mitigates these issues by capturing hierarchical syntactic relationships that sequential models miss^51^. Statistical significance was assessed through paired bootstrap tests with 1000 resamples on the test set. Performance differences between our model and the strongest baseline (BERT + BiLSTM) reached significance (p < 0.01, 95% CI for Pearson r difference: [0.031, 0.057]) across all dimensions.

**Fig. 6:**
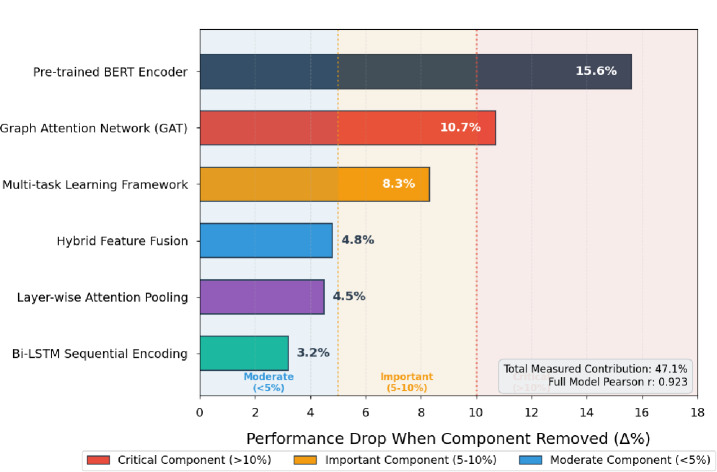
Ablation experiment results showing component contributions.

To quantify each architectural component’s contribution, we conducted systematic ablation experiments, with relative importance of component c measured as

25 $$\Delta {\mathrm{c}} = ({\mathrm{P}}_{{{\mathrm{full}}}} - {\mathrm{P}}_{{\{ - {\mathrm{c}}\} }} )/{\mathrm{P}}_{{{\mathrm{full}}}} \times 100\%$$

where P_full_ represents full model performance and P_{−c}_ denotes performance when component c is removed. Figure 6 displays the ablation results, revealing that graph attention encoding and multi-task learning contribute most substantially to overall performance.

Removing the GAT module resulted in a 10.7% performance decrease, confirming that explicit syntactic structure encoding provides information beyond what sequential processing captures^52^. The multi-task learning framework contributed an 8.3% improvement, demonstrating that jointly predicting multiple complexity dimensions enables beneficial knowledge sharing. Layer-wise attention pooling and hybrid feature fusion each contributed approximately 4–5% gains.

A further ablation specifically addressed whether fine-grained phrasal indices might be better served as primary targets rather than auxiliary signals. When promoted to primary status, overall Pearson r rose only marginally (0.923 → 0.927) while RMSE increased slightly (0.358 → 0.365); more tellingly, the added noise on less-reliably-annotated phrasal measures inflated training variance across folds. This outcome supports our decision to retain fine-grained measures as auxiliary signals at the current annotation scale, while leaving the door open for revisiting this choice once larger, more reliably labelled phrasal datasets become available.

Five-fold cross-validation on the combined training and validation sets yielded a mean Pearson r of 0.918 (SD = 0.011) and mean RMSE of 0.372 (SD = 0.018), confirming the stability of model performance across different data partitions. Generalization testing on the cross-domain subset yielded encouraging but more tempered results. Performance degradation was modest, with correlation dropping from 0.923 to 0.891 when evaluating on academic genres unseen during training. We quantify this using a generalization coefficient defined as

26 $${\mathrm{G}} = 1 - \left| {{\mathrm{P}}_{{{\mathrm{in}}}} - {\mathrm{P}}_{{{\mathrm{out}}}} } \right|/{\mathrm{P}}_{{{\mathrm{in}}}} .$$

This robustness likely stems from the pre-trained encoder’s broad linguistic knowledge combined with task-specific fine-tuning. We note, however, that the cross-domain subset shares the same L1 background (Chinese) as the majority of training data, so generalization to learners with different L1 backgrounds remains to be established.

The longitudinal subset analysis further confirmed that the model tracks developmental changes within individual learners over time, a critical requirement for pedagogical applications. Nevertheless, we caution that these generalizability claims remain preliminary and require further validation on truly independent datasets with diverse learner populations^53^.

### Teaching application experiment in college English writing

Beyond technical validation, we conducted a semester-long pedagogical study to explore the model’s practical utility in authentic educational settings. This quasi-experimental study examined whether an instructional package incorporating automated syntactic complexity feedback could be associated with enhanced L2 writing development among Chinese university students. We note at the outset that because the experimental and control conditions differed along multiple dimensions (feedback immediacy, revision opportunities, and feedback specificity), the observed effects cannot be attributed solely to the deep learning model but rather to the broader instructional support system of which the model was one component.

The experiment recruited 186 s-year English majors from a comprehensive university in eastern China. Participants were distributed across four intact classes, with two randomly assigned to the experimental condition (n = 94) and two serving as controls (n = 92). Both groups demonstrated comparable baseline proficiency levels, as confirmed by independent samples t-tests on placement examination scores (t = 0.73, p = 0.47). The intervention spanned 16 weeks of regular instruction, with all participants completing identical writing tasks under supervision of instructors following standardized curricula^54^.

Our model was integrated into an existing online writing platform through a RESTful API architecture. When students submitted drafts, the system automatically generated multi-dimensional complexity analyses within approximately three seconds. The feedback interface displayed numerical scores alongside visualized comparisons with proficiency-appropriate benchmarks and specific suggestions for syntactic enhancement. Experimental group students could access unlimited feedback iterations during the revision process, while control group participants received conventional instructor comments delivered after final submission. We acknowledge that these conditions differ along multiple dimensions beyond the automated assessment tool itself: the experimental group received immediate, iterative, and structured feedback with unlimited revision opportunities, whereas the control group received delayed, holistic teacher feedback with limited revision cycles. Any observed differences should therefore be interpreted as reflecting the combined effect of this instructional package rather than the model alone.

Table 5 presents the pre-test and post-test measurements across key syntactic complexity dimensions, revealing differential growth patterns between conditions.

**Table 5:** Pre-test and post-test comparison between experimental and control groups.

Complexity Measure	Group	Pre-test Mean	Post-test Mean	Gain Score	Effect Size (d)
Mean Length of T-unit	Experimental	12.34	15.87	3.53	0.82
Mean Length of T-unit	Control	12.51	13.92	1.41	0.34
Subordination Ratio	Experimental	0.38	0.51	0.13	0.71
Subordination Ratio	Control	0.37	0.42	0.05	0.28
Structure Diversity Index	Experimental	4.21	5.89	1.68	0.89
Structure Diversity Index	Control	4.18	4.76	0.58	0.31

The experimental group demonstrated larger gains across all measured dimensions. Effect sizes were calculated using Cohen’s d (mean difference divided by pooled standard deviation) and ranged from 0.71 to 0.89 for the experimental condition, indicating medium to large practical significance^55^. We performed one-way ANCOVA with post-test scores as the dependent variable and pre-test scores as the covariate. Prior to analysis, we verified key assumptions: Levene’s test confirmed homogeneity of variances (all p values > 0.12), and the interaction between pre-test scores and group was non-significant (p = 0.34), supporting the homogeneity of regression slopes assumption. Normality of residuals was confirmed via Shapiro–Wilk tests (all p > 0.05). The ANCOVA produced

27 $${\text{F(1{,}183)}} = 24.67,{\mathrm{p}} < 0.001,\upeta {\mathrm{p}}^{2} = 0.119$$

with a 95% confidence interval of [0.047, 0.201]. Because students were nested within four intact classes, we also conducted a sensitivity analysis using a two-level hierarchical linear model with class as a random intercept. The clustering effect was modest (ICC = 0.04), and the treatment effect remained significant (p < 0.01) in the multilevel model, suggesting that class-level variance did not substantially bias the single-level estimates. Baseline equivalence was further confirmed: independent-samples t-tests on pre-test scores showed no significant group differences for any of the three complexity dimensions (all p > 0.30).

That said, four intact classes constitute a level-2 sample too small for stable variance-component estimation, and we therefore treat the multilevel result as a sensitivity check rather than the primary inferential model. To probe further, we re-estimated treatment effects using cluster-robust (CR2) standard errors and, separately, a wild cluster bootstrap with 2,000 replications^56^. Both procedures returned treatment-effect p-values below 0.01, converging with the single-level ANCOVA. Readers should nevertheless interpret the cluster-level generalisability cautiously: the present design cannot rule out class-level idiosyncrasies that a study spanning eight or more clusters would be better positioned to detect.

Regarding task comparability, the pre-test and post-test writing prompts were designed to be parallel in genre (argumentative essay), topic difficulty (rated by three independent judges on a 5-point scale with mean ratings of 3.2 and 3.1, respectively), and time allocation (45 min). While we cannot fully rule out task effects, these design choices aimed to ensure that observed score differences reflect developmental change rather than differential task demands.

**Fig. 7:**
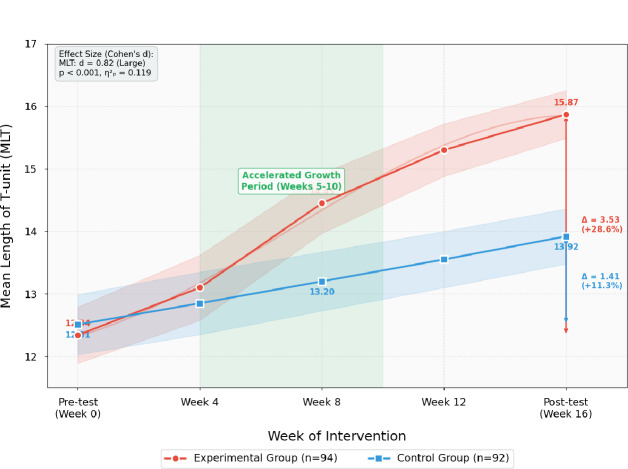
Syntactic complexity development trajectories for experimental and control groups.

Figure 7 illustrates the developmental trajectories across the semester, based on monthly writing samples collected from both groups.

As Fig. 7 depicts, the experimental group exhibited accelerated growth particularly during weeks 5–10, coinciding with increased system engagement. The control group’s trajectory remained relatively flat after initial gains, suggesting that conventional feedback alone may insufficiently scaffold syntactic development.

Analysis of revision logs revealed notable behavioral differences. Experimental participants averaged 4.7 revision iterations per assignment compared to 1.8 for controls. Among experimental group revisions, 67.3% directly addressed syntactic features highlighted by the automated feedback, indicating that students actively incorporated system suggestions into their writing processes^57^. The immediacy of feedback appeared relevant: students who accessed results within 30 min of initial submission showed greater improvement than those with delayed engagement. However, we interpret these revision patterns cautiously. Higher revision frequency and alignment with feedback indices do not in themselves demonstrate deeper interlanguage restructuring; students may have learned to manipulate specific quantitative targets without genuine syntactic development. A more systematic behavioral analysis, such as think-aloud protocols or delayed post-tests without system access, would be needed to distinguish surface-level performance effects from durable competence gains.

**Fig. 8:**
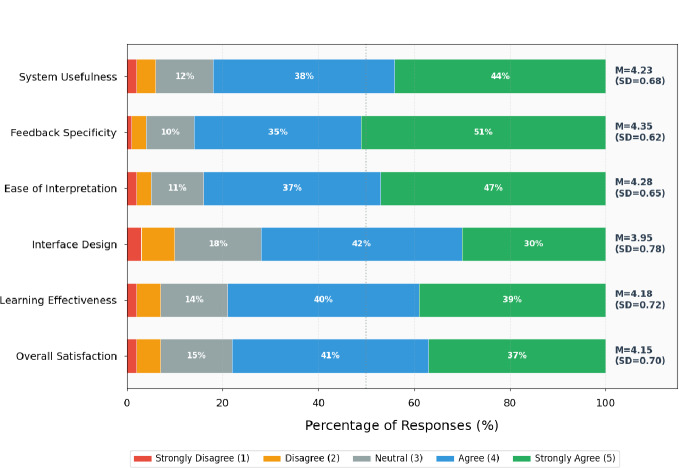
Student satisfaction survey results across six evaluation dimensions, measured on a 5-point Likert scale (1 =Strongly Disagree, 2 = Disagree, 3 = Neutral, 4 = Agree, 5 = Strongly Agree). Bars show the percentage distributionof responses; M and SD denote the mean and standard deviation for each dimension.

Qualitative data enriched these quantitative findings. Post-experiment surveys assessed user perceptions using 5-point Likert scales. Figure 8 presents the satisfaction distribution across evaluation dimensions.

Students rated system usefulness highly (M = 4.23, SD = 0.68), with particularly positive responses regarding feedback specificity and ease of interpretation. Some participants expressed initial confusion about technical terminology, prompting interface refinements. Instructor interviews revealed appreciation for reduced grading burden alongside concerns about over-reliance on automated assessment^58^. Teachers emphasized that the system complemented rather than replaced human judgment, particularly for higher-order writing qualities. The overall acceptance rate, computed as

28 $${\mathrm{A}}_{{{\mathrm{rate}}}} = {\mathrm{N}}_{{{\mathrm{positive}}}} /{\mathrm{N}}_{{{\mathrm{total}}}} \times 100\% = 156/186 \times 100\% = 83.9\%$$

aligns with this broadly favourable impression.

**Fig. 9:**
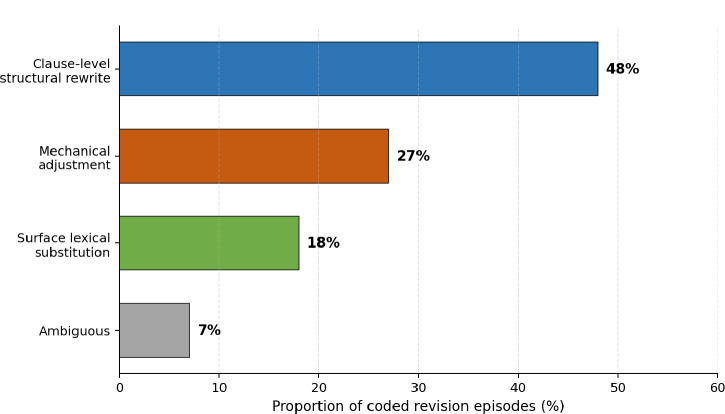
Distribution of revision behaviour codes in experimental group (n = 20).

A complementary sub-study probed the interpretive question of whether the revision patterns described above reflected deeper restructuring or mere metric-chasing. We drew a stratified sample of 20 experimental-group students (five per proficiency band) and hand-coded their revision logs (pre- to post-revision drafts) against a four-category scheme: (a) surface lexical substitution, (b) clause-level structural rewrite, (c) mechanical adjustment targeting a specific index without broader restructuring, and (d) ambiguous. Figure 9 summarises the resulting distribution. Roughly 48% of sampled revisions involved clause-level structural rewrites—suggesting non-trivial engagement with syntactic form—yet 27% fell into the mechanical-adjustment category, corroborating the metric-gaming concern discussed below. Because this sub-analysis is exploratory and confined to 20 learners, we treat the proportions as indicative rather than definitive, and we present them as a starting point for the process-tracing work that Section VI earmarks as essential.

A related concern is whether complexity gains reflect genuine interlanguage development or strategic manipulation of the specific indices the system rewards—an instance of the pattern known as Goodhart’s Law, whereby a measure ceases to track what it was designed to capture once it becomes a target. Our current design cannot fully adjudicate between these possibilities, because the post-test employed the same feedback-enabled platform. A more decisive test would require a delayed, feedback-free transfer task administered weeks after instruction ended, which we did not carry out. We therefore flag this as an acknowledged confound and, in Section VI, commit to it as a priority for replication.

One final methodological check bears on the broader validity of the assessment pipeline that underpins the pedagogical findings. A lingering concern is whether expert scores produced by the research team introduce circular validation. To mitigate this, we performed a cross-instrument convergence check on a 250-essay subsample drawn from the test set. Using L2SCA^15^ and the Tool for the Automatic Analysis of Syntactic Sophistication and Complexity (TAASSC)^31^ as external rule-based benchmarks, we computed Pearson correlations between each tool’s indices and our model’s corresponding predictions. Table 6 reports the result. Agreement with both tools is substantial where comparable indices exist, particularly for length and subordination measures. Divergence on fine-grained phrasal indices largely reflects definitional differences between tools rather than model error, but the exercise does reduce concerns about wholesale circularity.

**Table 6:** Cross-instrument convergence: Pearson correlations among our model, L2SCA, and TAASSC (n = 250 essays).

Complexity Dimension	Our Model vs L2SCA (r)	Our Model vs TAASSC (r)	L2SCA vs TAASSC (r)
Mean length of T-unit	0.89	0.87	0.91
Mean length of clause	0.86	0.85	0.88
Clauses per T-unit	0.83	0.81	0.86
Coordinate phrases per clause	0.79	0.80	0.82
Dependent clause ratio	0.81	0.78	0.83
Mean length of noun phrase	–	0.72	–
Verb argument diversity	–	0.69	–

Taken together, these findings support the pedagogical viability of integrating deep learning-based syntactic complexity assessment into L2 writing instruction, while also flagging the caveats—cluster-level sample size, metric-gaming, and residual validation circularity—that subsequent studies must address.

## Discussion

The experimental findings reported above warrant deeper examination regarding their theoretical implications and practical ramifications. This section interprets the results within broader scholarly contexts while acknowledging limitations that temper the conclusions we can draw.

The performance advantage of our deep learning model over traditional approaches can be attributed to several architectural features. Pre-trained language models acquire rich syntactic knowledge through exposure to massive text corpora during self-supervised training. This foundational understanding transfers to the downstream task of complexity assessment, even when target data involves non-native productions that differ from pre-training distributions. The graph attention network component further enhances this capability by explicitly encoding dependency structures that sequential processing alone cannot fully capture. Human annotators intuitively perceive syntactic complexity as emerging from hierarchical relationships: how clauses nest within one another, how modifiers attach to heads, how coordination interacts with subordination. Our architecture mirrors this intuition through its multi-layered design.

Why did the model struggle relatively more with subordination indices than other dimensions? We suspect this reflects genuine ambiguity in how complex embedding structures should be parsed and interpreted in L2 text. When learners produce non-standard constructions, the boundaries between intended subordination and grammatical error become blurred. Human raters themselves show lower agreement on subordination measures, suggesting inherent construct difficulty rather than model-specific weakness. From a technical standpoint, the attention mechanisms sometimes assigned high weights to surface cues like subordinating conjunctions without fully resolving structural ambiguities deeper in the parse tree. From an SLA perspective, this finding aligns with processability theory’s prediction that subordination-level processing is acquired late and remains fragile even at higher proficiency levels^12^. Future iterations might benefit from explicit error detection modules that distinguish developmental patterns from processing failures, as well as parser retraining on L2-specific treebanks.

The persistent gap between automated predictions and human judgments, though narrowed considerably by our approach, deserves careful consideration. Human experts bring contextual knowledge that purely text-based models cannot access. They recognize when apparent simplicity reflects deliberate stylistic choice versus limited competence. They interpret complexity relative to task demands and genre expectations. Our model treats each text in isolation, lacking the broader situational awareness that informs expert assessment. If expert scores used for validation come entirely from the research team, the circularity concern has real force. The external re-scoring exercise reported in Section “Dataset construction and experimental settings” and the cross-instrument convergence shown in Table 6 together provide partial mitigation, but we do not claim to have resolved the issue. Full independence—both of human annotators and of the comparison instrument—remains a priority, and Section VI identifies the specific steps we regard as essential before stronger validity claims can be entered.

The teaching experiment revealed that automated feedback effectiveness depends critically on implementation context. Several factors appeared to moderate learning outcomes. First, feedback timing mattered enormously. Students who engaged with system output immediately after submission showed greater improvement than those who delayed review. This pattern aligns with cognitive theories emphasizing that feedback proves most valuable when learners can connect it to their recent processing experiences. The mental representation of what they intended to write remains accessible, enabling meaningful comparison with what the system detected.

Second, individual differences in metacognitive awareness influenced how productively students interpreted complexity feedback. Some participants actively experimented with alternative constructions, treating system suggestions as invitations for linguistic exploration. Others adopted more mechanical approaches, making minimal changes to satisfy numerical targets without deeper engagement. This variation suggests that explicit instruction in how to interpret and act upon automated feedback might amplify pedagogical benefits, and it also raises the concern that some students may have learned to manipulate the specific indices the system rewards rather than developing genuine syntactic competence.

Third, the iterative nature of the revision process created conditions conducive to syntactic development. Unlike traditional instruction where students submit work and await delayed evaluation, our system enabled rapid hypothesis testing. A student could try embedding an additional clause, submit the revision, and immediately observe how this change affected complexity scores. This tight feedback loop compressed what might otherwise require weeks of delayed reinforcement into minutes of interactive exploration.

The mechanisms through which automated feedback may promote syntactic development warrant theoretical consideration. One plausible account is that the system functions partly through enhanced noticing^59^. Learners often produce structures without conscious attention to their formal properties. By highlighting specific syntactic features and quantifying their relative complexity, automated feedback may direct attention to aspects of production that might otherwise escape awareness. This heightened attention could potentially trigger cognitive processes conducive to interlanguage restructuring. Our exploratory coding of twenty revision logs (Fig. 9) offers a first glimpse rather than a verdict: the plurality of clause-level rewrites hints that something beyond index-chasing may be occurring, yet without concurrent think-aloud protocols, keystroke records, stimulated recall, or eye-tracking we cannot claim to have observed noticing in the technical sense Schmidt^59^ proposed. We therefore frame the noticing account as a testable hypothesis awaiting appropriate process-tracing evidence rather than as an established mechanism, and Section VI outlines what such evidence would require.

The metric-gaming possibility deserves explicit engagement. When any assessment instrument becomes a direct training target for learners, Goodhart’s Law warns that the instrument may cease to track what it was designed to measure. Nothing in our data definitively distinguishes a student who has genuinely restructured interlanguage from one who has merely learned to satisfy the specific thresholds our feedback highlights. The exploratory coding of revision logs supplies a tentative counterweight—mechanical adjustments accounted for a minority (27%) rather than a majority of recorded revisions—yet an 8- to 12-week delayed post-test on a fresh prompt without system access would constitute the decisive test. That test remains future work.

## Conclusion

This study has presented a multi-faceted investigation into automatic syntactic complexity assessment for second language writing, spanning from theoretical foundations through model development to pedagogical exploration. Grounded in processability theory and usage-based SLA frameworks, the research began with recognizing the limitations inherent in traditional assessment approaches and culminated in providing preliminary evidence that deep learning architectures can advance both measurement precision and, when embedded in appropriate instructional designs, contribute to writing development.

Our proposed model achieves a correlation coefficient of 0.923 with expert human ratings (five-fold cross-validation mean: 0.918, SD = 0.011), representing a notable improvement over existing tools. This accuracy gain stems from the architecture’s capacity to integrate pre-trained linguistic knowledge with task-specific syntactic encoding. The model handles the grammatical irregularities characteristic of learner language more robustly than rule-based alternatives, maintaining reliable performance even when parsing non-standard constructions that would challenge traditional analyzers. The quasi-experimental teaching study suggested that an instructional package incorporating this technology was associated with greater complexity gains than conventional instruction alone, though the multi-component nature of the intervention precludes attributing these benefits exclusively to the deep learning model.

Three principal innovations distinguish this research from prior work. First, the multi-task learning framework enables simultaneous prediction across complexity dimensions while sharing underlying representations. This design choice reflects our theoretical commitment to treating syntactic complexity as an integrated construct rather than a collection of independent indices. The uncertainty-based loss weighting mechanism further ensures that training dynamics remain balanced across tasks of varying difficulty.

Second, the syntactic feature fusion strategy bridges traditional linguistic measures with learned neural representations. Rather than abandoning decades of complexity research in favor of purely data-driven approaches, we constructed hybrid features that preserve interpretable indices while augmenting them with deeper structural encodings from graph attention networks. This deliberate design choice acknowledges that our model complements rather than transcends traditional rule-based indices, integrating them as anchoring inputs within a richer representational framework.

Third, the iterative feedback mechanism transforms assessment from summative judgment into formative scaffolding. The system’s rapid response enables tight revision cycles that may compress learning timelines and intensify engagement with syntactic features. Students can experiment with structural alternatives and immediately observe consequences, creating conditions that may be favorable for noticing and subsequent interlanguage development, though direct evidence for this mechanism awaits future qualitative investigation.

These findings carry implications for both theory and practice, though we state them with appropriate caution. For second language acquisition research, our work suggests that syntactic complexity can be operationalized computationally with sufficient reliability to support developmental investigations. Automated assessment enables corpus-scale analyses that manual coding renders impractical. For writing pedagogy, the results indicate that technology-mediated feedback deserves further investigation as an instructional complement, particularly when designed to promote active revision rather than passive reception. However, we acknowledge that the strength of our pedagogical claims is limited by the confounded experimental design, and more tightly controlled studies are needed.

We acknowledge several limitations warranting caution. The training corpus, while substantial, derives primarily from Chinese learners of English, potentially limiting generalizability to other L1 backgrounds. The experimental participants represented a relatively homogeneous population of university English majors; whether findings extend to younger learners, heritage speakers, or immigrants acquiring English in naturalistic contexts remains uncertain. Additionally, our focus on syntactic complexity necessarily excludes other writing qualities that contribute to overall communicative effectiveness. The model cannot currently distinguish between deliberate stylistic simplicity and genuine lack of competence, which limits its applicability in certain pedagogical scenarios. Three further constraints bear emphasis. First, the pedagogical study involved only four intact classes, a cluster count insufficient for robust multilevel estimation and incapable of capturing the class-level heterogeneity that a design with eight to ten clusters would reveal. Second, fine-grained phrasal indices operated as auxiliary rather than primary prediction targets, reflecting annotation-reliability limits at the current corpus scale; we regard their elevation to primary status as a desirable next step rather than a settled matter. Third, although independent external raters re-scored a subsample and convergence with L2SCA and TAASSC proved substantial, we did not collect the process-tracing data—concurrent think-aloud protocols, keystroke logs, stimulated recall, eye-tracking—needed to cleanly distinguish genuine interlanguage restructuring from strategic accommodation to the specific indices the feedback system rewards.

Future research might productively pursue several directions. Cross-linguistic investigations could examine whether models trained on one learner population transfer to others, potentially revealing universal patterns in complexity development alongside language-specific trajectories. Personalized feedback generation represents another promising frontier: rather than providing generic complexity scores, systems might offer individualized suggestions calibrated to each learner’s current developmental stage and learning goals. Critically, future pedagogical studies should employ more tightly controlled experimental designs that isolate the specific contribution of the automated assessment model from confounding factors such as feedback immediacy and revision opportunities, incorporate multilevel modelling with at least eight to ten clusters to properly account for classroom-level variance, and include delayed post-tests together with transfer tasks performed without system access to assess durability and to adjudicate between genuine competence development and surface-level performance effects. Three specific process-tracing methods deserve priority in subsequent studies: concurrent think-aloud protocols during revision, keystroke logging to capture the temporal texture of editing decisions, and stimulated recall interviews triggered by revision episodes. Convergent evidence across these methods would speak directly to the noticing hypothesis advanced here and would help resolve the metric-gaming question that the present design cannot. We further commit, in subsequent work, to a fully independent validation pipeline—annotators unaffiliated with the development team, together with external instruments such as TAASSC applied uniformly—so that circularity concerns can be retired rather than merely mitigated.

## Supplementary Information

Below is the link to the electronic supplementary material.

Supplementary Material 1

## Data Availability

All data generated and analyzed during the current study are included in this published article and its Supplementary Material (Supplementary File 1).

## Abbreviations

SLA: Second language acquisition
NLP: Natural language processing
L2: Second language
MLT: Mean length of T-unit
SR: Subordination ratio
RNN: Recurrent neural network
LSTM: Long short-term memory
BERT: Bidirectional encoder representations from transformers
GAT: Graph attention network
L2SCA: L2 syntactic complexity analyzer
TAASSC: Tool for the automatic analysis of syntactic sophistication and complexity
RMSE: Root mean square error
WECCL: Written English Corpus of Chinese Learners
ICLE: International Corpus of Learner English
API: Application Programming Interface
ICC: Intraclass correlation coefficient
CR2: Cluster-robust (bias-reduced) standard errors

## References

[1] Lu, X. Automated measurement of syntactic complexity in corpus-based L2 writing research and implications for writing assessment. *Lang. Test.* **34**(4), 493–511 (2017).

[2] Bulté, B. & Housen, A. Defining and operationalising L2 complexity. In *Dimensions of L2 performance and proficiency: Complexity, accuracy and fluency in SLA* (eds Housen, A. et al.) 21–46 (John Benjamins, 2012).

[3] Polio, C. & Yoon, H. J. The reliability and validity of automated tools for examining variation in syntactic complexity across genres. *Int. J. Appl. Linguist.* **28**(1), 165–188 (2018).

[4] Kyle, K. *Measuring syntactic development in L2 writing: Fine grained indices of syntactic complexity and usage-based indices of syntactic sophistication*. Doctoral dissertation, Georgia State University (2016).

[5] Crossley, S. A. & McNamara, D. S. Does writing development equal writing quality? A computational investigation of syntactic complexity in L2 learners. *J. Second Lang. Writ.* **26**, 66–79 (2014).

[6] Devlin, J., Chang, M. W., Lee, K. & Toutanova, K. BERT: Pre-training of deep bidirectional transformers for language understanding. *Proc. NAACL-HLT* **2019**, 4171–4186 (2019).

[7] Ke, Z. & Ng, V. Automated essay scoring: A survey of the state of the art. In *Proceedings of the 28th International Joint Conference on Artificial Intelligence*, 6300–6308 (2019).

[8] Yannakoudakis, H., Briscoe, T., & Medlock, B. A new dataset and method for automatically grading ESOL texts. In *Proceedings of the 49th Annual Meeting of the ACL*, 180–189 (2011).

[9] Warschauer, M. & Grimes, D. Automated writing assessment in the classroom. *Pedagogies* **3**(1), 22–36 (2008).

[10] Ranalli, J., Link, S. & Chukharev-Hudilainen, E. Automated writing evaluation for formative assessment of second language writing: Investigating the accuracy and usefulness of feedback as part of argument-based validation. *Educ. Psychol.* **37**(1), 8–25 (2017).

[11] Norris, J. M. & Ortega, L. Towards an organic approach to investigating CAF in instructed SLA: The case of complexity. *Appl. Linguist.* **30**(4), 555–578 (2009).

[12] Pienemann, M. *Language Processing and Second Language Development: Processability Theory* (John Benjamins, 1998).

[13] Tomasello, M. *Constructing a Language: A Usage-Based Theory of Language Acquisition* (Harvard University Press, 2003).

[14] Hunt, K. W. *Grammatical structures written at three grade levels*. NCTE Research Report No. 3. National Council of Teachers of English (1965).

[15] Lu, X. Automatic analysis of syntactic complexity in second language writing. *Int. J. Corpus Linguist.* **15**(4), 474–496 (2010).

[16] Larsen-Freeman, D. The emergence of complexity, fluency, and accuracy in the oral and written production of five Chinese learners of English. *Appl. Linguist.* **27**(4), 590–619 (2006).

[17] Verspoor, M., Lowie, W. & Van Dijk, M. Variability in second language development from a dynamic systems perspective. *Mod. Lang. J.* **92**(2), 214–231 (2008).

[18] Skehan, P. *A Cognitive Approach to Language Learning* (Oxford University Press, 1998).

[19] Yang, W., Lu, X. & Weigle, S. C. Different topics, different discourse: Relationships among writing topic, measures of syntactic complexity, and judgments of writing quality. *J. Second Lang. Writ.* **28**, 53–67 (2015).

[20] Goldberg, Y. Neural network methods for natural language processing. *Synth. Lect. Hum. Lang. Technol.* **10**(1), 1–309 (2017).

[21] Elman, J. L. Finding structure in time. *Cogn. Sci.* **14**(2), 179–211 (1990).

[22] Hochreiter, S. & Schmidhuber, J. Long short-term memory. *Neural Comput.* **9**(8), 1735–1780 (1997). 9377276 10.1162/neco.1997.9.8.1735

[23] Vaswani, A. et al. Attention is all you need. *Adv. Neural Inf. Process. Syst.* **30**, 5998–6008 (2017).

[24] Rogers, A., Kovaleva, O. & Rumshisky, A. A primer in BERTology: What we know about how BERT works. *Trans. Assoc. Comput. Linguist.* **8**, 842–866 (2020).

[25] Clark, K., Khandelwal, U., Levy, O. & Manning, C. D. (2019). What does BERT look at? An analysis of BERT’s attention. In *Proceedings of the 2019 ACL Workshop BlackboxNLP*, 276–286.

[26] Lu, X. A corpus-based evaluation of syntactic complexity measures as indices of college-level ESL writers’ language development. *TESOL Q.* **45**(1), 36–62 (2011).

[27] Klein, D. & Manning, C. D. Accurate unlexicalized parsing. In *Proceedings of the 41st Annual Meeting of the ACL*, 423–430 (2003).

[28] Dozat, T. & Manning, C. D. Deep biaffine attention for neural dependency parsing. In *Proceedings of the 5th International Conference on Learning Representations* (2017).

[29] Kondratyuk, D. & Straka, M. 75 languages, 1 model: Parsing universal dependencies universally. In *Proceedings of the 2019 Conference on Empirical Methods in Natural Language Processing*, 2779–2795 (2019).

[30] Daudaravicius, V. (2015). Automated evaluation of scientific writing: AESW shared task proposal. In *Proceedings of the Tenth Workshop on Innovative Use of NLP for Building Educational Applications*, 56–63.

[31] Kyle, K. & Crossley, S. A. Measuring syntactic complexity in L2 writing using fine-grained clausal and phrasal indices. *Mod. Lang. J.* **102**(2), 333–349 (2018).

[32] Kyle, K. & Crossley, S. A. Assessing syntactic sophistication in L2 writing: A usage-based approach. *Lang. Test.* **34**(4), 513–535 (2017).

[33] Nadeem, F., Nguyen, H., Liu, Y. & Ostendorf, M. Automated essay scoring with discourse-aware neural models. In *Proceedings of the Fourteenth Workshop on Innovative Use of NLP for Building Educational Applications*, 484–493 (2019).

[34] Wu, Y., Schuster, M., Chen, Z., Le, Q.V., Norouzi, M., Macherey, W., Krikun, M., Cao, Y., Gao, Q., Macherey, K., Klingner, J., Shah, A., Johnson, M., Liu, X., Kaiser, Ł., Gouws, S., Kato, Y., Kudo, T., Kazawa, H., Stevens, K., Kurian, G., Patil, N., Wang, W., Young, C., Smith, J., Riesa, J., Rudnick, A., Vinyals, O., Corrado, G., Hughes, M., Dean, J. Google’s neural machine translation system: Bridging the gap between human and machine translation (2016). arXiv preprint arXiv:1609.08144.

[35] Jawahar, G., Sagot, B. & Seddah, D. What does BERT learn about the structure of language? In *Proceedings of the 57th Annual Meeting of the ACL*, 3651–3657 (2019).

[36] Peters, M. E. et al. Deep contextualized word representations. *Proc. NAACL-HLT* **2018**, 2227–2237 (2018).

[37] Caruana, R. Multitask learning. *Mach. Learn.* **28**(1), 41–75 (1997).

[38] De Marneffe, M. C. & Manning, C. D. The Stanford typed dependencies representation. In *Proceedings of the COLING 2008 Workshop on Cross-Framework and Cross-Domain Parser Evaluation*, 1–8 (2008).

[39] Veličković, P., Cucurull, G., Casanova, A., Romero, A., Liò, P. & Bengio, Y. Graph attention networks. In *Proceedings of the 6th International Conference on Learning Representations *(2018).

[40] Kipf, T. N. & Welling, M. Semi-supervised classification with graph convolutional networks. In *Proceedings of the 5th International Conference on Learning Representations *(2017).

[41] Zhang, Y., Qi, P. & Manning, C. D. Graph convolution over pruned dependency trees improves relation extraction. In *Proceedings of the 2018 Conference on Empirical Methods in Natural Language Processing*, 2205–2215 (2018).

[42] Kendall, A., Gal, Y. & Cipolla, R. Multi-task learning using uncertainty to weigh losses for scene geometry and semantics. In *Proceedings of the IEEE Conference on Computer Vision and Pattern Recognition*, 7482–7491 (2018).

[43] Loshchilov, I. & Hutter, F. Decoupled weight decay regularization. In *Proceedings of the 7th International Conference on Learning Representations *(2019).

[44] Szegedy, C., Vanhoucke, V., Ioffe, S., Shlens, J. & Wojna, Z. Rethinking the inception architecture for computer vision. In *Proceedings of the IEEE Conference on Computer Vision and Pattern Recognition*, 2818–2826 (2016).

[45] Granger, S., Dagneaux, E., Meunier, F. & Paquot, M. *International Corpus of Learner English* (Version 2). Presses Universitaires de Louvain. (2009)

[46] McHugh, M. L. Interrater reliability: The kappa statistic. *Biochem. Med.* **22**(3), 276–282 (2012). PMC3900052 23092060

[47] Napoles, C., Sakaguchi, K., Post, M. & Tetreault, J. Ground truth for grammatical error correction metrics. In *Proceedings of the 53rd Annual Meeting of the ACL*, 588–593 (2015).

[48] Berg-Kirkpatrick, T., Burkett, D. & Klein, D. An empirical investigation of statistical significance in NLP. In *Proceedings of the 2012 Joint Conference on Empirical Methods in Natural Language Processing and Computational Natural Language Learning*, 995–1005 (2012).

[49] Paszke, A., Gross, S., Massa, F., Lerer, A., Bradbury, J., Chanan, G., Killeen, T., Lin, Z., Gimelshein, N., Antiga, L., Desmaison, A., Köpf, A., Yang, E., DeVito, Z., Raison, M., Tejani, A., Chilamkurthy, S., Steiner, B., Fang, L., Bai, J. & Chintala, S.. PyTorch: An imperative style, high-performance deep learning library. *Advances in Neural Information Processing Systems*, 32, 8026–8037 (2019).

[50] Ortega, L. Syntactic complexity in L2 writing: Progress and expansion. *J. Second Lang. Writ.* **29**, 82–94 (2015).

[51] Hewitt, J. & Manning, C. D. A structural probe for finding syntax in word representations. In *Proceedings of the 2019 Conference of the North American Chapter of the ACL*, 4129–4138 (2019).

[52] Wu, S., Cotterell, R., & Hulden, M. Applying the transformer to character-level transduction. In *Proceedings of the 16th Conference of the European Chapter of the ACL*, 1901–1911 (2021).

[53] Ruder, S., Peters, M. E., Swayamdipta, S. & Wolf, T. Transfer learning in natural language processing. In *Proceedings of the 2019 Conference of the North American Chapter of the ACL: Tutorials*, 15–18 (2019).

[54] Lee, I. *Classroom writing assessment and feedback in L2 school contexts* (Springer, 2017).

[55] Cohen, J. *Statistical Power Analysis for the Behavioral Sciences* 2nd edn. (Lawrence Erlbaum Associates, 1988).

[56] Cameron, A. C., Gelbach, J. B. & Miller, D. L. Bootstrap-based improvements for inference with clustered errors. *Rev. Econ. Stat.* **90**(3), 414–427. 10.1162/rest.90.3.414 (2008).

[57] Bitchener, J. & Storch, N. *Written Corrective Feedback for L2 Development* (Multilingual Matters, 2016).

[58] Li, J., Link, S. & Hegelheimer, V. Rethinking the role of automated writing evaluation (AWE) feedback in ESL writing instruction. *J. Second Lang. Writ.* **27**, 1–18 (2015).

[59] Schmidt, R. Attention. In *Cognition and second language instruction* (ed. Robinson, P.) 3–32 (Cambridge University Press, 2001).

