# Supplementary material for: A deep learning-based model for automatic syntactic complexity assessment in L2 English writing: development and pedagogical application
Source: Sci Rep. 2026 May 29;16:24568. doi: 10.1038/s41598-026-53282-0 (PMC13454429; doi:10.1038/s41598-026-53282-0)
Supplement: Supplementary file 1 [file 41598_2026_53282_MOESM1_ESM.docx]

# Supplementary Material

## A Deep Learning-Based Model for Automatic Syntactic Complexity Assessment in L2 English Writing: Development and Pedagogical Application

**Supplementary Table S1. Detailed Hyperparameter Configuration**

| Hyperparameter | Value |
| --- | --- |
| Pre-trained Model | BERT-base-uncased |
| Vocabulary Size | 30,522 |
| Hidden Dimension | 768 |
| Number of Transformer Layers | 12 |
| Number of Attention Heads | 12 |
| Maximum Sequence Length | 512 |
| Batch Size | 32 |
| Learning Rate (Base) | 2e-5 |
| Warmup Steps | 1,000 |
| Total Training Steps | 26,425 |
| Weight Decay (λ) | 0.01 |
| Dropout Rate | 0.1 |
| Label Smoothing (ε) | 0.1 |
| GAT Layers | 3 |
| GAT Attention Heads | 4 |
| GAT Hidden Dimension | 256 |
| BiLSTM Hidden Dimension | 512 |
| BiLSTM Layers | 2 |
| Early Stopping Patience | 5 epochs |
| Optimizer | AdamW |
| Adam β₁ | 0.9 |
| Adam β₂ | 0.999 |
| Adam ε | 1e-8 |
| Gradient Clipping Max Norm | 1.0 |
| Random Seed | 42 |

**Supplementary Table S2. Annotation Guidelines for Syntactic Complexity Dimensions**

| Dimension | Index | Scoring Criteria | Scale |
| --- | --- | --- | --- |
| Length Measures | Mean Length of T-unit (MLT) | Average number of words per T-unit; calculated automatically after T-unit segmentation | Continuous (words) |
| Length Measures | Mean Length of Clause (MLC) | Average number of words per clause; includes both independent and dependent clauses | Continuous (words) |
| Amount Measures | Clauses per T-unit (C/T) | Number of clauses divided by number of T-units | Continuous (ratio) |
| Amount Measures | Coordinate Phrases per Clause (CP/C) | Number of coordinate phrases divided by total clauses | Continuous (ratio) |
| Subordination | Dependent Clause Ratio (DCR) | Number of dependent clauses divided by total clauses | Continuous (0–1) |
| Subordination | Maximum Embedding Depth (MED) | Deepest level of clause embedding in each T-unit, averaged across text | Continuous (levels) |
| Diversity | Syntactic Structure Types (SST) | Count of unique syntactic construction types per text sample | Discrete (count) |
| Diversity | Verb Pattern Variation (VPV) | Number of different verb argument structure types | Discrete (count) |
| Overall | Holistic Complexity Rating | Expert judgment integrating all dimensions | 1–6 Likert |

**Supplementary Table S3. Inter-Rater Reliability for Each Complexity Dimension**

| Dimension | Cohen’s κ | Pearson r | Exact Agreement (%) | Adjacent Agreement (%) |
| --- | --- | --- | --- | --- |
| Mean Length of T-unit | 0.91 | 0.96 | 94.2 | 99.1 |
| Mean Length of Clause | 0.89 | 0.95 | 92.7 | 98.6 |
| Clauses per T-unit | 0.85 | 0.92 | 88.4 | 96.3 |
| Coordinate Phrases per Clause | 0.82 | 0.90 | 85.6 | 95.1 |
| Dependent Clause Ratio | 0.79 | 0.88 | 82.3 | 93.8 |
| Maximum Embedding Depth | 0.77 | 0.86 | 80.1 | 92.4 |
| Syntactic Structure Types | 0.84 | 0.91 | 87.2 | 95.7 |
| Verb Pattern Variation | 0.81 | 0.89 | 84.5 | 94.2 |
| Holistic Complexity Rating | 0.83 | 0.91 | 86.8 | 95.4 |
| Overall Average | 0.83 | 0.91 | 86.9 | 95.6 |

**Supplementary Table S4. Detailed Performance Metrics Across Proficiency Levels**

| Model | Proficiency | RMSE | Pearson r | MAE | Spearman ρ |
| --- | --- | --- | --- | --- | --- |
| L2SCA | A2 | 0.912 | 0.681 | 0.743 | 0.664 |
| L2SCA | B1 | 0.834 | 0.724 | 0.689 | 0.708 |
| L2SCA | B2 | 0.821 | 0.738 | 0.671 | 0.721 |
| L2SCA | C1 | 0.856 | 0.706 | 0.712 | 0.689 |
| BiLSTM | A2 | 0.689 | 0.752 | 0.561 | 0.738 |
| BiLSTM | B1 | 0.614 | 0.798 | 0.503 | 0.781 |
| BiLSTM | B2 | 0.597 | 0.812 | 0.488 | 0.796 |
| BiLSTM | C1 | 0.631 | 0.784 | 0.519 | 0.769 |
| BERT-base | A2 | 0.562 | 0.821 | 0.447 | 0.808 |
| BERT-base | B1 | 0.489 | 0.858 | 0.391 | 0.843 |
| BERT-base | B2 | 0.471 | 0.869 | 0.378 | 0.856 |
| BERT-base | C1 | 0.513 | 0.842 | 0.412 | 0.829 |
| Ours (Full) | A2 | 0.412 | 0.901 | 0.328 | 0.889 |
| Ours (Full) | B1 | 0.347 | 0.929 | 0.276 | 0.918 |
| Ours (Full) | B2 | 0.331 | 0.936 | 0.264 | 0.924 |
| Ours (Full) | C1 | 0.378 | 0.914 | 0.301 | 0.903 |

**Supplementary Table S5. Detailed Ablation Study Results**

| Configuration | RMSE | Pearson r | Δ Performance (%) | Length r | Subordination r | Diversity r |
| --- | --- | --- | --- | --- | --- | --- |
| Full Model | 0.358 | 0.923 | — | 0.931 | 0.908 | 0.930 |
| − GAT Module | 0.401 | 0.897 | −10.7 | 0.903 | 0.876 | 0.912 |
| − Multi-task Learning | 0.389 | 0.906 | −8.3 | 0.914 | 0.887 | 0.917 |
| − Layer-wise Attention | 0.378 | 0.912 | −4.8 | 0.920 | 0.894 | 0.922 |
| − Hybrid Feature Fusion | 0.381 | 0.910 | −4.2 | 0.918 | 0.892 | 0.920 |
| − Label Smoothing | 0.371 | 0.916 | −2.7 | 0.924 | 0.899 | 0.925 |
| − Learning Rate Warmup | 0.374 | 0.914 | −3.1 | 0.922 | 0.897 | 0.923 |
| − BiLSTM Layer | 0.386 | 0.908 | −5.6 | 0.916 | 0.889 | 0.919 |
| − Uncertainty Weighting | 0.383 | 0.909 | −5.2 | 0.917 | 0.891 | 0.919 |
| − GAT − Multi-task | 0.432 | 0.874 | −18.1 | 0.886 | 0.849 | 0.887 |

**Supplementary Table S6. Cross-Domain Generalization Results by Genre**

| Genre | N | RMSE | Pearson r | Generalization Coefficient (G) |
| --- | --- | --- | --- | --- |
| Argumentative Essay | 156 | 0.372 | 0.916 | 0.962 |
| Narrative Essay | 112 | 0.381 | 0.909 | 0.957 |
| Expository Essay | 98 | 0.394 | 0.898 | 0.951 |
| Summary Writing | 87 | 0.408 | 0.887 | 0.943 |
| Letter/Email | 70 | 0.421 | 0.876 | 0.935 |
| Overall Cross-domain | 523 | 0.391 | 0.891 | 0.965 |

**Supplementary Table S7. Participant Demographic Information**

| Characteristic | Experimental Group (n = 94) | Control Group (n = 92) | Test Statistic | p value |
| --- | --- | --- | --- | --- |
| Gender (Female/Male) | 61/33 | 58/34 | χ² = 0.12 | 0.73 |
| Age (Mean ± SD) | 20.3 ± 1.1 | 20.1 ± 1.2 | t = 1.14 | 0.26 |
| CET-4 Score (Mean ± SD) | 487.6 ± 42.3 | 491.2 ± 39.8 | t = 0.58 | 0.56 |
| Placement Exam Score (Mean ± SD) | 72.4 ± 8.6 | 73.1 ± 8.2 | t = 0.73 | 0.47 |
| Years of English Study (Mean ± SD) | 11.2 ± 1.4 | 11.5 ± 1.3 | t = 1.42 | 0.16 |
| Self-rated Writing Proficiency (1–5) | 2.87 ± 0.74 | 2.91 ± 0.71 | t = 0.37 | 0.71 |

**Supplementary Table S8. Detailed Pre-test and Post-test Results Across All Complexity Measures**

| Measure | Group | Pre-test Mean (SD) | Post-test Mean (SD) | Gain Score | 95% CI of Gain | t | p | Cohen’s d |
| --- | --- | --- | --- | --- | --- | --- | --- | --- |
| MLT | Experimental | 12.34 (3.21) | 15.87 (3.58) | 3.53 | [2.89, 4.17] | 8.42 | <0.001 | 0.82 |
| MLT | Control | 12.51 (3.18) | 13.92 (3.34) | 1.41 | [0.82, 2.00] | 3.67 | <0.001 | 0.34 |
| MLC | Experimental | 8.76 (2.14) | 11.23 (2.47) | 2.47 | [1.94, 3.00] | 7.56 | <0.001 | 0.76 |
| MLC | Control | 8.82 (2.08) | 9.64 (2.21) | 0.82 | [0.34, 1.30] | 2.89 | 0.005 | 0.27 |
| C/T | Experimental | 1.42 (0.31) | 1.78 (0.35) | 0.36 | [0.27, 0.45] | 6.84 | <0.001 | 0.73 |
| C/T | Control | 1.40 (0.29) | 1.52 (0.32) | 0.12 | [0.04, 0.20] | 2.51 | 0.014 | 0.26 |
| CP/C | Experimental | 0.28 (0.11) | 0.37 (0.12) | 0.09 | [0.06, 0.12] | 5.47 | <0.001 | 0.64 |
| CP/C | Control | 0.27 (0.10) | 0.31 (0.11) | 0.04 | [0.01, 0.07] | 2.12 | 0.037 | 0.24 |
| DCR | Experimental | 0.38 (0.09) | 0.51 (0.10) | 0.13 | [0.10, 0.16] | 7.21 | <0.001 | 0.71 |
| DCR | Control | 0.37 (0.08) | 0.42 (0.09) | 0.05 | [0.02, 0.08] | 2.78 | 0.007 | 0.28 |
| MED | Experimental | 1.86 (0.52) | 2.54 (0.58) | 0.68 | [0.53, 0.83] | 6.93 | <0.001 | 0.75 |
| MED | Control | 1.84 (0.49) | 2.07 (0.53) | 0.23 | [0.09, 0.37] | 2.64 | 0.010 | 0.29 |
| SST | Experimental | 4.21 (1.34) | 5.89 (1.42) | 1.68 | [1.32, 2.04] | 8.87 | <0.001 | 0.89 |
| SST | Control | 4.18 (1.28) | 4.76 (1.36) | 0.58 | [0.24, 0.92] | 2.74 | 0.007 | 0.31 |
| VPV | Experimental | 3.54 (1.12) | 4.87 (1.24) | 1.33 | [1.01, 1.65] | 7.64 | <0.001 | 0.83 |
| VPV | Control | 3.48 (1.08) | 3.92 (1.14) | 0.44 | [0.14, 0.74] | 2.46 | 0.016 | 0.28 |

**Supplementary Table S9. ANCOVA Results for Between-Group Comparisons**

| Measure | F(1,183) | p | Partial η² | Observed Power |
| --- | --- | --- | --- | --- |
| Mean Length of T-unit | 24.67 | <0.001 | 0.119 | 0.998 |
| Mean Length of Clause | 19.84 | <0.001 | 0.098 | 0.993 |
| Clauses per T-unit | 17.52 | <0.001 | 0.087 | 0.988 |
| Coordinate Phrases per Clause | 12.36 | <0.001 | 0.063 | 0.939 |
| Dependent Clause Ratio | 15.89 | <0.001 | 0.080 | 0.978 |
| Maximum Embedding Depth | 14.23 | <0.001 | 0.072 | 0.963 |
| Syntactic Structure Types | 27.41 | <0.001 | 0.130 | 0.999 |
| Verb Pattern Variation | 22.18 | <0.001 | 0.108 | 0.997 |

**Supplementary Table S10. Monthly Developmental Trajectory Data**

| Timepoint | Measure | Experimental Mean (SD) | Control Mean (SD) |
| --- | --- | --- | --- |
| Week 0 (Pre-test) | MLT | 12.34 (3.21) | 12.51 (3.18) |
| Week 4 | MLT | 13.12 (3.28) | 12.83 (3.22) |
| Week 8 | MLT | 14.56 (3.41) | 13.21 (3.27) |
| Week 12 | MLT | 15.34 (3.52) | 13.64 (3.31) |
| Week 16 (Post-test) | MLT | 15.87 (3.58) | 13.92 (3.34) |
| Week 0 (Pre-test) | DCR | 0.38 (0.09) | 0.37 (0.08) |
| Week 4 | DCR | 0.41 (0.09) | 0.38 (0.08) |
| Week 8 | DCR | 0.46 (0.10) | 0.40 (0.09) |
| Week 12 | DCR | 0.49 (0.10) | 0.41 (0.09) |
| Week 16 (Post-test) | DCR | 0.51 (0.10) | 0.42 (0.09) |
| Week 0 (Pre-test) | SST | 4.21 (1.34) | 4.18 (1.28) |
| Week 4 | SST | 4.58 (1.36) | 4.31 (1.30) |
| Week 8 | SST | 5.24 (1.39) | 4.52 (1.32) |
| Week 12 | SST | 5.67 (1.41) | 4.63 (1.34) |
| Week 16 (Post-test) | SST | 5.89 (1.42) | 4.76 (1.36) |

**Supplementary Table S11. System Engagement Metrics**

| Metric | Experimental Group Mean (SD) | Control Group Mean (SD) |
| --- | --- | --- |
| Total Submissions | 47.3 (12.6) | 18.4 (6.2) |
| Revision Iterations per Assignment | 4.7 (1.8) | 1.8 (0.7) |
| Average Time Between Submission and Feedback Access (min) | 8.4 (14.2) | N/A |
| Percentage of Revisions Targeting Syntax | 67.3% | 23.1% |
| Average Session Duration (min) | 34.6 (11.3) | 21.2 (8.7) |
| Feedback Views per Assignment | 6.2 (2.4) | N/A |
| Percentage of Students Accessing Within 30 min | 72.3% | N/A |
| Total Writing Words Produced | 14,287 (3,214) | 8,462 (2,187) |

**Supplementary Table S12. Student Satisfaction Survey Items and Results**

| Item No. | Survey Item | Mean | SD | Strongly Disagree (%) | Disagree (%) | Neutral (%) | Agree (%) | Strongly Agree (%) |
| --- | --- | --- | --- | --- | --- | --- | --- | --- |
| 1 | The system provides useful feedback on my writing | 4.23 | 0.68 | 0.0 | 3.2 | 11.7 | 44.7 | 40.4 |
| 2 | The feedback is easy to understand | 3.98 | 0.81 | 1.1 | 5.3 | 17.0 | 45.7 | 30.9 |
| 3 | The system helps me identify syntactic weaknesses | 4.31 | 0.64 | 0.0 | 2.1 | 8.5 | 46.8 | 42.6 |
| 4 | I can improve my writing based on the feedback | 4.18 | 0.72 | 0.0 | 3.2 | 14.9 | 42.6 | 39.4 |
| 5 | The response time is acceptable | 4.52 | 0.59 | 0.0 | 1.1 | 5.3 | 34.0 | 59.6 |
| 6 | I would recommend this system to other students | 4.07 | 0.79 | 1.1 | 4.3 | 14.9 | 44.7 | 35.1 |
| 7 | The visual feedback display is clear | 4.14 | 0.74 | 0.0 | 4.3 | 12.8 | 45.7 | 37.2 |
| 8 | The system motivates me to revise my writing | 4.36 | 0.67 | 0.0 | 2.1 | 9.6 | 39.4 | 48.9 |
| 9 | Technical terms in feedback are understandable | 3.72 | 0.93 | 2.1 | 9.6 | 22.3 | 41.5 | 24.5 |
| 10 | Overall satisfaction with the system | 4.19 | 0.71 | 0.0 | 3.2 | 12.8 | 44.7 | 39.4 |

**Supplementary Table S13. Training Convergence Metrics**

| Epoch | Training Loss | Validation Loss | Validation Pearson r | Learning Rate |
| --- | --- | --- | --- | --- |
| 1 | 1.284 | 0.987 | 0.724 | 4.0e-6 |
| 2 | 0.823 | 0.712 | 0.798 | 8.0e-6 |
| 3 | 0.614 | 0.578 | 0.841 | 1.2e-5 |
| 4 | 0.487 | 0.491 | 0.869 | 1.6e-5 |
| 5 | 0.398 | 0.438 | 0.886 | 2.0e-5 |
| 6 | 0.341 | 0.402 | 0.897 | 1.9e-5 |
| 7 | 0.296 | 0.381 | 0.908 | 1.8e-5 |
| 8 | 0.261 | 0.367 | 0.914 | 1.6e-5 |
| 9 | 0.234 | 0.358 | 0.919 | 1.4e-5 |
| 10 | 0.212 | 0.354 | 0.921 | 1.2e-5 |
| 11 | 0.194 | 0.352 | 0.923 | 1.0e-5 |
| 12 | 0.179 | 0.354 | 0.922 | 7.8e-6 |
| 13 | 0.168 | 0.358 | 0.921 | 5.7e-6 |

Note: Training stopped at epoch 16 (early stopping triggered at epoch 11 + patience 5). Best checkpoint selected at epoch 11.

**Supplementary Table S14. Computational Efficiency Comparison**

| Model | Training Time (hours) | Inference Time per Sample (ms) | GPU Memory (GB) | Total Parameters (M) |
| --- | --- | --- | --- | --- |
| L2SCA | N/A | 1,240 | N/A (CPU) | N/A |
| BiLSTM | 2.3 | 28 | 4.2 | 12.7 |
| BERT-base | 5.1 | 45 | 8.6 | 109.5 |
| RoBERTa-base | 5.4 | 47 | 8.8 | 124.6 |
| BERT + BiLSTM | 6.2 | 52 | 10.3 | 117.8 |
| Ours (w/o GAT) | 6.8 | 56 | 11.1 | 121.3 |
| Ours (Full Model) | 8.0 | 63 | 12.4 | 146.1 |

**Supplementary Table S15. Error Analysis by Error Type in L2 Writing**

| Error Type | Frequency in Test Set (%) | Full Model RMSE | BERT-base RMSE | L2SCA RMSE |
| --- | --- | --- | --- | --- |
| Subject-Verb Agreement | 18.4 | 0.367 | 0.512 | 0.891 |
| Tense Errors | 15.7 | 0.354 | 0.498 | 0.867 |
| Article Misuse | 14.2 | 0.341 | 0.476 | 0.823 |
| Run-on Sentences | 11.8 | 0.398 | 0.547 | 0.934 |
| Fragment Sentences | 9.6 | 0.412 | 0.563 | 0.978 |
| Word Order Errors | 8.3 | 0.389 | 0.534 | 0.912 |
| Missing Subordinators | 7.1 | 0.378 | 0.521 | 0.897 |
| Comma Splices | 6.4 | 0.392 | 0.541 | 0.945 |
| Other Errors | 8.5 | 0.361 | 0.507 | 0.856 |

**Supplementary Table S16. Correlation Matrix Among Complexity Dimensions (Test Set)**

| Dimension | MLT | MLC | C/T | CP/C | DCR | MED | SST | VPV |
| --- | --- | --- | --- | --- | --- | --- | --- | --- |
| MLT | 1.000 | 0.834 | 0.712 | 0.523 | 0.687 | 0.654 | 0.598 | 0.576 |
| MLC | 0.834 | 1.000 | 0.489 | 0.467 | 0.534 | 0.512 | 0.478 | 0.456 |
| C/T | 0.712 | 0.489 | 1.000 | 0.612 | 0.823 | 0.789 | 0.634 | 0.621 |
| CP/C | 0.523 | 0.467 | 0.612 | 1.000 | 0.434 | 0.398 | 0.512 | 0.487 |
| DCR | 0.687 | 0.534 | 0.823 | 0.434 | 1.000 | 0.856 | 0.612 | 0.589 |
| MED | 0.654 | 0.512 | 0.789 | 0.398 | 0.856 | 1.000 | 0.578 | 0.567 |
| SST | 0.598 | 0.478 | 0.634 | 0.512 | 0.612 | 0.578 | 1.000 | 0.823 |
| VPV | 0.576 | 0.456 | 0.621 | 0.487 | 0.589 | 0.567 | 0.823 | 1.000 |

Note: All correlations significant at p < 0.001.

**Supplementary Table S17. Feedback Interface Feature Description**

| Feature | Description | Implementation |
| --- | --- | --- |
| Complexity Dashboard | Multi-dimensional radar chart displaying scores across all 8 indices | D3.js visualization |
| Proficiency Benchmark | Color-coded comparison with CEFR-level norms (A2–C1) | Reference corpus statistics |
| Sentence Highlighting | Individual sentences color-coded by complexity level | CSS gradient mapping |
| Suggestion Panel | Specific syntactic enhancement recommendations | Rule-template + neural generation |
| History Tracker | Visualization of complexity score changes across revisions | Line chart with version comparison |
| T-unit Segmentation View | Interactive display of parsed T-unit boundaries | Dependency parse output |
| Clause Tree Viewer | Hierarchical visualization of clause embedding structure | Tree layout rendering |
| Export Function | PDF report generation for instructor review | Server-side PDF rendering |

**Supplementary Code S1. Core Model Architecture (PyTorch)**

```
import torch
import torch.nn as nn
from transformers import BertModel
from torch_geometric.nn import GATConv

class SyntacticComplexityModel(nn.Module):
    def __init__(self, config):
        super(SyntacticComplexityModel, self).__init__()

        # Pre-trained BERT encoder
        self.bert = BertModel.from_pretrained(config.bert_model_name)

        # Learnable layer weights for multi-layer fusion
        self.layer_weights = nn.Parameter(
            torch.ones(config.num_hidden_layers) / config.num_hidden_layers
        )

        # Graph Attention Network for syntactic encoding
        self.gat_layers = nn.ModuleList([
            GATConv(
                in_channels=config.hidden_size if i == 0 else config.gat_hidden,
                out_channels=config.gat_hidden,
                heads=config.gat_heads,
                concat=True if i < config.num_gat_layers - 1 else False,
                dropout=config.dropout
            )
            for i in range(config.num_gat_layers)
        ])

        # Bidirectional LSTM for sequential processing
        self.bilstm = nn.LSTM(
            input_size=config.hidden_size,
            hidden_size=config.lstm_hidden,
            num_layers=config.lstm_layers,
            batch_first=True,
            bidirectional=True,
            dropout=config.dropout
        )

        # Feature fusion layer
        fused_dim = config.lstm_hidden * 2 + config.gat_hidden + config.trad_feature_dim
        self.fusion_layer = nn.Sequential(
            nn.Linear(fused_dim, config.fusion_hidden),
            nn.LayerNorm(config.fusion_hidden),
            nn.GELU(),
            nn.Dropout(config.dropout)
        )

        # Multi-task prediction heads
        self.length_head = self._build_prediction_head(config.fusion_hidden, 128, 1)
        self.subordination_head = self._build_prediction_head(config.fusion_hidden, 64, 1)
        self.diversity_head = self._build_prediction_head(config.fusion_hidden, 64, 1)
        self.overall_head = self._build_prediction_head(config.fusion_hidden, 128, 1)

        # Uncertainty parameters for loss weighting
        self.log_vars = nn.Parameter(torch.zeros(4))

    def _build_prediction_head(self, input_dim, hidden_dim, output_dim):
        return nn.Sequential(
            nn.Linear(input_dim, hidden_dim),
            nn.GELU(),
            nn.Dropout(0.1),
            nn.Linear(hidden_dim, hidden_dim // 2),
            nn.GELU(),
            nn.Linear(hidden_dim // 2, output_dim)
        )

    def forward(self, input_ids, attention_mask, edge_index, trad_features):
        # BERT encoding with all hidden states
        outputs = self.bert(
            input_ids=input_ids,
            attention_mask=attention_mask,
            output_hidden_states=True
        )

        # Weighted layer fusion
        hidden_states = torch.stack(outputs.hidden_states[1:], dim=0)
        weights = torch.softmax(self.layer_weights, dim=0)
        fused_bert = torch.einsum('l,lbsh->bsh', weights, hidden_states)

        # GAT encoding over dependency graph
        gat_input = fused_bert.mean(dim=1)  # Document-level
        x = gat_input
        for gat_layer in self.gat_layers:
            x = gat_layer(x, edge_index)
            x = torch.relu(x)
        gat_output = x

        # BiLSTM processing
        lstm_output, _ = self.bilstm(fused_bert)
        lstm_pooled = lstm_output.mean(dim=1)

        # Hybrid feature fusion
        combined = torch.cat([lstm_pooled, gat_output, trad_features], dim=-1)
        fused = self.fusion_layer(combined)

        # Multi-task predictions
        length_pred = self.length_head(fused)
        subord_pred = self.subordination_head(fused)
        diversity_pred = self.diversity_head(fused)
        overall_pred = torch.sigmoid(self.overall_head(fused))

        return {
            'length': length_pred,
            'subordination': subord_pred,
            'diversity': diversity_pred,
            'overall': overall_pred
        }

    def compute_loss(self, predictions, targets):
        losses = []
        task_keys = ['length', 'subordination', 'diversity', 'overall']

        for i, key in enumerate(task_keys):
            task_loss = nn.MSELoss()(predictions[key].squeeze(), targets[key])
            precision = torch.exp(-self.log_vars[i])
            weighted_loss = precision * task_loss + self.log_vars[i]
            losses.append(weighted_loss)

        return sum(losses)
```

**Supplementary Code S2. Training Pipeline**

```
from torch.optim import AdamW
from torch.optim.lr_scheduler import LambdaLR
import math

def get_cosine_warmup_scheduler(optimizer, warmup_steps, total_steps):
    def lr_lambda(step):
        if step < warmup_steps:
            return step / warmup_steps
        progress = (step - warmup_steps) / (total_steps - warmup_steps)
        return max(0.0, 0.5 * (1.0 + math.cos(math.pi * progress)))
    return LambdaLR(optimizer, lr_lambda)

def train_model(model, train_loader, val_loader, config):
    optimizer = AdamW(
        model.parameters(),
        lr=config.learning_rate,
        weight_decay=config.weight_decay,
        betas=(0.9, 0.999),
        eps=1e-8
    )

    total_steps = len(train_loader) * config.num_epochs
    scheduler = get_cosine_warmup_scheduler(
        optimizer, config.warmup_steps, total_steps
    )

    best_val_loss = float('inf')
    patience_counter = 0

    for epoch in range(config.num_epochs):
        model.train()
        epoch_loss = 0.0

        for batch in train_loader:
            optimizer.zero_grad()

            predictions = model(
                input_ids=batch['input_ids'].to(config.device),
                attention_mask=batch['attention_mask'].to(config.device),
                edge_index=batch['edge_index'].to(config.device),
                trad_features=batch['trad_features'].to(config.device)
            )

            targets = {k: batch[f'target_{k}'].to(config.device)
                      for k in ['length', 'subordination', 'diversity', 'overall']}

            loss = model.compute_loss(predictions, targets)
            loss.backward()

            torch.nn.utils.clip_grad_norm_(model.parameters(), max_norm=1.0)

            optimizer.step()
            scheduler.step()
            epoch_loss += loss.item()

        # Validation
        val_loss, val_metrics = evaluate(model, val_loader, config)

        print(f"Epoch {epoch+1}/{config.num_epochs} | "
              f"Train Loss: {epoch_loss/len(train_loader):.4f} | "
              f"Val Loss: {val_loss:.4f} | "
              f"Val Pearson r: {val_metrics['pearson_r']:.4f}")

        # Early stopping
        if val_loss < best_val_loss:
            best_val_loss = val_loss
            patience_counter = 0
            torch.save(model.state_dict(), config.best_model_path)
        else:
            patience_counter += 1
            if patience_counter >= config.patience:
                print(f"Early stopping at epoch {epoch+1}")
                break

    return model
```

**Supplementary Code S3. Traditional Feature Extraction**

```
import spacy
from collections import Counter

class TraditionalFeatureExtractor:
    def __init__(self):
        self.nlp = spacy.load("en_core_web_sm")

    def extract_features(self, text):
        doc = self.nlp(text)

        t_units = self._segment_t_units(doc)
        clauses = self._identify_clauses(doc)
        dep_clauses = self._identify_dependent_clauses(doc)

        features = {}

        # Length measures
        features['mlt'] = (sum(len(tu) for tu in t_units) / len(t_units)
                          if t_units else 0)
        features['mlc'] = (sum(len(cl) for cl in clauses) / len(clauses)
                          if clauses else 0)

        # Amount measures
        features['c_per_t'] = (len(clauses) / len(t_units)
                              if t_units else 0)
        features['cp_per_c'] = self._count_coord_phrases(doc) / max(len(clauses), 1)

        # Subordination measures
        features['dcr'] = (len(dep_clauses) / len(clauses)
                          if clauses else 0)
        features['med'] = self._max_embedding_depth(doc)

        # Diversity measures
        features['sst'] = len(self._get_structure_types(doc))
        features['vpv'] = len(self._get_verb_patterns(doc))

        return features

    def _segment_t_units(self, doc):
        t_units = []
        current_tu = []
        for sent in doc.sents:
            for token in sent:
                current_tu.append(token)
                if token.dep_ == 'ROOT' and current_tu:
                    t_units.append(current_tu)
                    current_tu = []
            if current_tu:
                t_units.append(current_tu)
                current_tu = []
        return t_units

    def _identify_clauses(self, doc):
        clauses = []
        for token in doc:
            if token.dep_ in ('ROOT', 'ccomp', 'xcomp', 'advcl',
                             'relcl', 'acl', 'csubj'):
                clause_tokens = [t for t in token.subtree]
                clauses.append(clause_tokens)
        return clauses if clauses else [[t for t in doc]]

    def _identify_dependent_clauses(self, doc):
        dep_labels = {'ccomp', 'xcomp', 'advcl', 'relcl', 'acl', 'csubj'}
        return [t for t in doc if t.dep_ in dep_labels]

    def _max_embedding_depth(self, doc):
        max_depth = 0
        for token in doc:
            depth = 0
            current = token
            while current.head != current:
                if current.dep_ in ('ccomp', 'xcomp', 'advcl', 'relcl', 'acl'):
                    depth += 1
                current = current.head
            max_depth = max(max_depth, depth)
        return max_depth

    def _count_coord_phrases(self, doc):
        return sum(1 for t in doc if t.dep_ == 'conj')

    def _get_structure_types(self, doc):
        patterns = set()
        for sent in doc.sents:
            pattern = tuple(t.dep_ for t in sent if t.dep_ != 'punct')
            patterns.add(pattern)
        return patterns

    def _get_verb_patterns(self, doc):
        patterns = set()
        for token in doc:
            if token.pos_ == 'VERB':
                children_deps = tuple(sorted(c.dep_ for c in token.children
                                            if c.dep_ != 'punct'))
                patterns.add((token.lemma_, children_deps))
        return patterns
```

**Supplementary Table S18. Software and Library Versions**

| Software/Library | Version |
| --- | --- |
| Python | 3.10.12 |
| PyTorch | 2.0.1 |
| Transformers (Hugging Face) | 4.30.2 |
| PyTorch Geometric | 2.3.1 |
| spaCy | 3.5.3 |
| NumPy | 1.24.3 |
| SciPy | 1.10.1 |
| scikit-learn | 1.2.2 |
| CUDA Toolkit | 11.8 |
| cuDNN | 8.6.0 |
| Operating System | Ubuntu 22.04 LTS |

**Supplementary Table S19. Statistical Tests for Assumption Checking**

| Test | Variable/Group | Statistic | p value | Assumption Met |
| --- | --- | --- | --- | --- |
| Shapiro-Wilk (Normality) | MLT Pre-test Experimental | W = 0.987 | 0.412 | Yes |
| Shapiro-Wilk (Normality) | MLT Pre-test Control | W = 0.984 | 0.367 | Yes |
| Shapiro-Wilk (Normality) | DCR Pre-test Experimental | W = 0.981 | 0.298 | Yes |
| Shapiro-Wilk (Normality) | DCR Pre-test Control | W = 0.979 | 0.264 | Yes |
| Shapiro-Wilk (Normality) | SST Pre-test Experimental | W = 0.976 | 0.213 | Yes |
| Shapiro-Wilk (Normality) | SST Pre-test Control | W = 0.974 | 0.187 | Yes |
| Levene’s (Homogeneity) | MLT Pre-test | F = 0.34 | 0.562 | Yes |
| Levene’s (Homogeneity) | DCR Pre-test | F = 0.18 | 0.674 | Yes |
| Levene’s (Homogeneity) | SST Pre-test | F = 0.27 | 0.604 | Yes |
| Box’s M (Homogeneity of Covariance) | All measures combined | M = 18.42 | 0.231 | Yes |
| Regression Homogeneity (ANCOVA) | MLT | F = 1.23 | 0.269 | Yes |
| Regression Homogeneity (ANCOVA) | DCR | F = 0.87 | 0.352 | Yes |
| Regression Homogeneity (ANCOVA) | SST | F = 1.06 | 0.305 | Yes |
